# Current clinical landscape of oncolytic viruses as novel cancer immunotherapeutic and recent preclinical advancements

**DOI:** 10.3389/fimmu.2022.953410

**Published:** 2022-08-25

**Authors:** Chae-Ok Yun, JinWoo Hong, A-Rum Yoon

**Affiliations:** ^1^ Department of Bioengineering, College of Engineering, Hanyang University, Seoul, South Korea; ^2^ Institute of Nano Science and Technology (INST), Hanyang University, Seoul, South Korea; ^3^ Hanyang Institute of Bioscience and Biotechnology (HY-IBB), Hanyang University, Seoul, South Korea; ^4^ GeneMedicine CO., Ltd., Seoul, South Korea

**Keywords:** oncolytic virus, clinical research, combination, radiation, chemotherapy, immunotherapy

## Abstract

Oncolytic viruses (OVs) have been gaining attention in the pharmaceutical industry as a novel immunotherapeutic and therapeutic adjuvant due to their ability to induce and boost antitumor immunity through multiple mechanisms. First, intrinsic mechanisms of OVs that enable exploitation of the host immune system (*e.g.*, evading immune detection) can nullify the immune escape mechanism of tumors. Second, many types of OVs have been shown to cause direct lysis of tumor cells, resulting in an induction of tumor-specific T cell response mediated by release of tumor-associated antigens and danger signal molecules. Third, armed OV-expressing immune stimulatory therapeutic genes could be highly expressed in tumor tissues to further improve antitumor immunity. Last, these OVs can inflame cold tumors and their microenvironment to be more immunologically favorable for other immunotherapeutics. Due to these unique characteristics, OVs have been tested as an adjuvant of choice in a variety of therapeutics. In light of these promising attributes of OVs in the immune-oncology field, the present review will examine OVs in clinical development and discuss various strategies that are being explored in preclinical stages for the next generation of OVs that are optimized for immunotherapy applications.

## 1 Introduction

The last decade has seen considerable success of immune checkpoint inhibitors (ICI) and chimeric antigen receptor (CAR)-T cells that has highlighted immuno-oncology (IO) (1–4). Although both ICI and CAR-T cell therapy led to complete tumor regression and durable remission in a small subset of cancer patients, a larger fraction of the patients did not respond or showed limited response to these immunotherapeutics (5). Detailed examination of these poor responders to immunotherapy led to characterization of immunologically ‘cold’ tumors that possess low density of tumor-infiltrating lymphocytes and a highly immunosuppressive microenvironment (6, 7). Global pharmaceutical companies have been exploring various strategies to overcome the limited efficacy of immunotherapeutics against such poorly responding tumors.

To this end, oncolytic viruses (OVs) have garnered the attention of biopharmaceutical industries since the US Food and Drug Administration (FDA)- and European Medicines Agency (EMA)-approved the first OVs, Imlygic, in 2015. Both preclinical and clinical data of Imlygic, as well as numerous other OVs, have shown that OVs can warm immunologically cold tumors to improve overall antitumor immune response of various immunotherapeutics drugs (8). OVs possess several unique features that are beneficial for cancer immunotherapy applications, and these attributes cannot be mimicked by other conventional cancer therapeutics. In particular, OVs selectively propagate in and eradicate cancer cells through a domino-like cascading infection and subsequent lysis of tumor cells (9, 10), leading to generation of tumor lysates, pathogen-associated molecular patterns (PAMPs), damage-associated molecular patterns (DAMPs), and tumor-associated antigens (TAA) as well as increasing production of various cytokines and chemokines, such as type I interferons (IFNs) (11). These byproducts of the oncolytic process can augment various aspects of the antitumor immune (both innate and adaptive) response, such as TAA presentation by antigen-presenting cells (APCs), subsequent induction of tumor-specific T cell response, and immune activation of the tumor microenvironment (12, 13). Other noteworthy attributes of OVs are their strong abscopal effect leading to regression of distant metastatic tumors and establishment of tumor-specific immune memory that can confer protection against tumor recurrence/relapse (14). Furthermore, arming OVs with immune stimulatory genes (*e.g.*, cytokines, chemokines, co-stimulators, and modalities that can nullify negative immune regulators like immune checkpoints) can further improve the induction of tumor-specific immune response and restore immune surveillance function in the tumor microenvironment (15–19).

**Figure 1:**
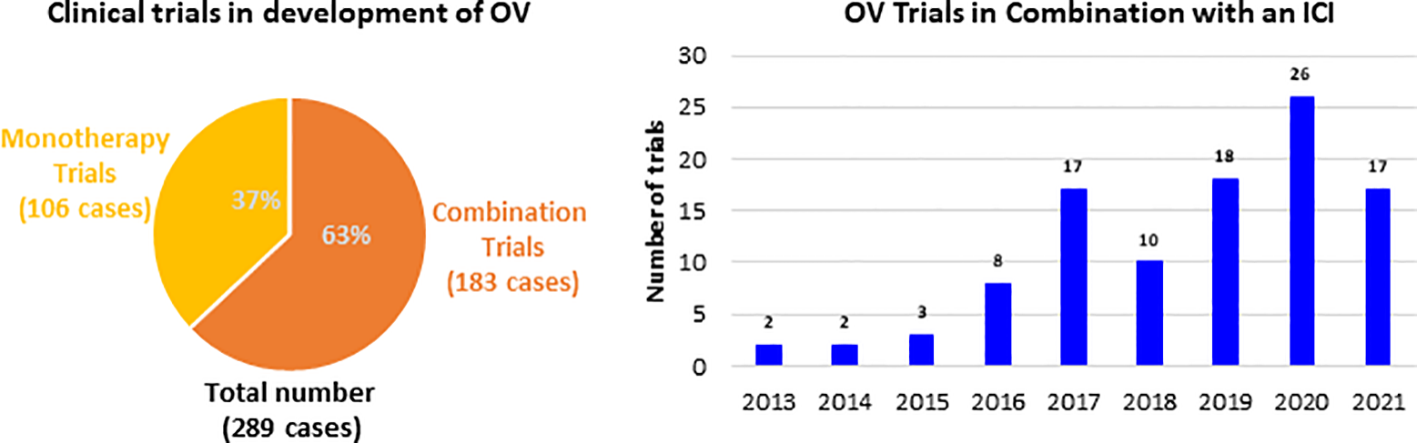
Clinical trials investigating OV and number of clinical trials combining OV with ICI.

These immune boosting properties of OVs are being actively explored both alone for therapy and in combination with other immunotherapeutics in clinical landscape. The therapeutic strategies with OV range from monotherapies to combination of other cancer therapies, including traditional cancer treatments and also other immunotherapies. Since 2013 the majority of clinical trials developing an OV were combination trials (183 out of 289), whereby an OV was administered in conjunction with another therapy (**Figure 1**). Among all the combination trials, the most common modality administered in combination with an OV is ICI, as more than 105 trials have been conducted. The trial start year distribution of these trials and it has grown over the past 8 years, and this growth is expected to continue. Further, much of the preliminary data forecast that OVs are likely to be an integral part of cancer immunotherapy in the near future.

There are many different types of OVs that had been investigated in clinical trials, but three viruses, oncolytic adenovirus (oAd), oncolytic herpes simplex virus (oHSV), oncolytic vaccinia virus (oVV), have the longest history and highest number of clinical trials conducted to date. This review will focus on preclinical and clinical development of these three OVs. Further, some of the other types of OVs, like oncolytic reovirus, oncolytic measles virus (oMV) and oncolytic picornaviruses, which are under active clinical development and where detailed and recent clinical studies are available will also be highlighted in the review. We explore the emergence and evolution of these OVs in preclinical and clinical landscapes and their role in advancement and understanding of IO. The review also discusses groundbreaking innovations and breakthroughs in OV application as both stand-alone and combination regimens to improve antitumor immunity, demonstrating that OVs could be widely adopted across different standard care options as promising adjuvant.

## 2 Current clinical trial landscape

### 2.1 Oncolytic adenovirus

Adenovirus was one of the earliest gene therapy vectors to be investigated in clinical trial (first human trial dates back to 1993) (20), and its clinical safety has been evaluated and documented extensively. Oncorine (which is similar to ONYX-015) was the first OV to be approved for clinical use in China in 2005, predating the US FDA and EMA approval of oHSV and Imlygic by a decade. Oncorine often is noted as a good testament to the extensive and historical development of oncolytic adenovirus (oAd) in the clinic (21). Major strengths and advantages of oAds rely on their ability to induce strong antitumor immune response (16, 22–24), anti-angiogenic effects (25, 26), high transgene expression, and synergistic anticancer effects in conjunction with conventional cancer therapies (9, 10, 27–29). Additionally, facile viral production at high titer makes the oAd production process economically advantageous (21). In lieu of these attributes, oAds are the most frequently used OV in clinical trials, accounting for ~42% of all trials (40 of 96 clinical trials, as presented in **Table 1**).

**Table 1:** Oncolytic adenoviruses tested in current clinical trials.

Oncolytic adenovirus	Tumor Selectivity	Route of Administration	Combination	Indication	Phases	NCT Number	Sponsor/Collaborator	Status
**CG0070**	E2F promoter	intravesical injection	none	Bladder Cancer	Phase 2	NCT02143804	Cold Genesys, Inc.	Withdrawn
			none		Phase 2	NCT02365818		Completed
			none		Phase 2/3	NCT01438112		Terminated
**DNX2401** **(Ad5-Δ24-RGD)**	24-base pair deletion in the E1A gene	Intratumoral injection	none	Brain Cancer	Phase 1	NCT03178032	DNAtrix, Inc.	Recruiting
		Intratumoral injection	Temozolomide		Phase 1	NCT01956734		Completed
		Intratumoral Injection	INF		Phase 1	NCT02197169		Completed
		Intratumoral Injection	pembrolizumab		Phase 2	NCT02798406		Active, not recruiting
		Intra-Arterial Injection	Conventional Surgery	Brain Cancer	Phase 1	NCT03896568	M.D. Anderson Cancer Center, National Cancer Institute	Recruiting
**DNX2440** **(Ad5-Δ24-RGD/OX40L)**	24-base pair deletion in the E1A gene	stereotactical injection	none	Brain Cancer	Phase 1	NCT03714334	DNAtrix, Inc.	Recruiting
**LOAd703** **(oAd/CD40L and 4-BBL)**	24-base pair deletion in the E1A gene	image-guided intratumoral injection	none	Pancreatic Adenocarcinoma, Ovarian Cancer, Biliary Carcinoma, Colorectal Cancer	Phase 1/2	NCT03225989	Lokon Pharma AB	Recruiting
		Intratumoral Injection	gemcitabine, paclitaxel, atezolizumab	Pancreatic Cancer	Phase 1/2	NCT02705196		Recruiting
		Intratumoral injection	atezolizumab	Malignant Melanoma	Phase 1/2	NCT04123470		Not yet recruiting
**Enadenotucirev (ColoAd1; chimeric Ad11p/Ad3)**	Unknown^1^	intratumoral injection or Intravenous Infusion	none	Colon Cancer, Non-small Cell Lung Cancer, Bladder Cancer, Renal Cell Carcinoma	Phase 1	NCT02053220	PsiOxus Therapeutics Ltd	Completed
		Intratumoral Injection	Capecitabine, Radiation	Locally Advanced Rectal Cancer	Phase 1	NCT03916510		Recruiting
**NG-641** **(ColoAd1-FAP/CD3 bispecific FAP-Tac)**		intratumoral injection or Intravenous Infusion	none	Metastatic Cancer, Epithelial Tumor	Phase 1	NCT04053283		Not yet recruiting
**NG-350** **(ColoAd1-CD40 mAb)**		Intravesical Injection	none	Metastatic Cancer, Epithelial Tumor	Phase 1	NCT03852511		Recruiting
**ONCOS-102** **(oAd/GMCSF)**	24-base pair deletion in the E1A gene serotype 3 knob	intratumoral injection or Intravenous Infusion	none	Malignant Solid Tumour	Phase 1	NCT01598129	Targovax	Completed
		Intratumoral injection	Cyclophosphamide, Pembrolizumab	Melanoma	Phase 1	NCT03003676		Recruiting
		intratumoral injection	Pemetrexed/cisplatin (carboplatin), Cyclophosphamide	Pleural Mesothelioma	Phase 1/2	NCT02879669		Active, not recruiting
		intratumoral injection	DCVac/Pca, Cyclophosphamide	Prostate Cancer	Phase 1/2	NCT03514836	Sotio a.s.	Recruiting
**VCN-01** **(oAd/HA)**	E2F promoter regulating E1A gene	intravenous injection	Durvalumab	Head and Neck Squamous Cell Carcinoma,	Phase 1	NCT03799744	VCN Biosciences, S.L.	Recruiting
		Intravenous Injection	Gemcitabinem, Abraxane	Pancreatic cancer	Phase 1	NCT02045602		Active, not recruiting
**Ad5-yCD/mutTKSR39rep-ADP**	replication-competent adenovirus type 5 containing a yeast cytosine deaminase(yCD)/mutant sr39 herpes simplex virus thymidine kinase fusion (yCD/mutTKsr39) gene(E1) and the 11.6 kDa adenovirus death protein (ADP) gene(E3)	Intratumoral injection	none	Non-small Cell Lung Cancer	Phase 1	NCT03029871	Henry Ford Health System	Withdrawn
**Ad5-yCD/mutTKSR39rep-hIL12**		Intraprostatic Injection	none	Prostate Cancer	Phase 1	NCT02555397	Henry Ford Health System Baylor College of Medicine	Recruiting
**Ad5-yCD/mutTKSR39rep-hIL12** **CGTG-102** **(oAd/GMCSF)**		Intratumoral Injection	none	Metastatic Pancreatic Cancer	Phase 1	NCT03281382		Recruiting
	24-base pair deletion in the E1A gene	Intratumoral Injection	none	Tumors, Solid Tumors	Phase 1	NCT01437280		Withdrawn
**CadVEC** **(oAd/PDL1)**	24-base pair deletion in the E1A gene	Intratumoral injection	HER2- specific autologous CAR T	Bladder Cancer, Head and Neck Squamous Cell Carcinoma, Cancer of the Salivary Gland, Lung Cancer, Breast Cancer, Gastric Cancer, Esophageal Cancer, Colorectal Cancer, Pancreatic Adenocarcinoma	Phase 1	NCT03740256	Baylor College of Medicine Erasmus Medical Center, VU University Medical Center	Not yet recruiting
**delta-24-RGD**	24-base pair deletion in the E1A gene	Intracerebral infusion by convection enhanced delivery	none	Brain Tumor	Phase 1/2	NCT01582516		Completed
**OBP-301**	human telomerase reverse transcriptase gene (hTERT) promoter.	intratumoral injection	none	Melanoma	Phase 2	NCT03190824	Syneos Health, Oncolys BioPharma Inc	Active, not recruiting
**ORCA-010**	24-base pair deletion in the E1A gene	intratumoral injection	none	Prostate Cancer	Phase 1/2	NCT04097002	Orca Therapeutics B.V.	Not yet recruiting
**ICOVIR** **(Ad-DM-E2F-K-Delta24-RGD)**	24-base pair deletion in the E1A gene and E2F promoter	endovenous injection	none	Melanoma	Phase 1	NCT01864759	Institut Català d'Oncologia	Completed
**ICOVIR**	24-base pair deletion in the E1A gene	intravenous injection	MSC as a delivery tool	Solid Tumors	Phase 1/2	NCT01844661	Hospital Universitario Niño Jesús	Completed
**CRAd-Survivin-pk7**	human survivin promoter	After neurosurgical resection, NSC-CRAd-S-pk7 was injected into the walls of the resection cavity	NSC as a delivery tool	Brain Cancer	Phase 1	NCT03072134	Northwestern University	Recruiting

1. Enadenociturev (previously ColoAd1) is a chimera derived from laboratory setting. Unlike other oAds that are based on naturally occurring human serotype 5 Ad, the precise biological mechanism behind the cancer specificity of enadenociturev and its derivatives have not been published. There are several probable mechanisms that may explain the cancer specificity of enadenociturev provided in Discussion section of the original paper (30).

Despite of commercialization of Oncorine and its yearly growth in total usage in China, it has failed to demonstrate sufficient therapeutic benefit as a single agent for refractory solid tumors (21, 31). One of the likely explanations of the limited efficacy of Oncorine is the deletion of the adenoviral E1B 55 kDa gene, which endows the oAd with cancer specificity but restricts its overall viral replication capacity (32). Since the preliminary clinical trials with ONYX-015 in the 1990s, significant technological advancements have been witnessed in vector design and construction. In detail, majority of the newer human serotype 5 oAd constructs, like ONCOS-102, LoAd703, TILT-123, ORCA-010, CG0070, under active clinical development contain intact E1B 55 kDa gene to circumvent the attenuation in viral replication capacity, and rather employ different genetic engineering strategies like cancer-specific promoter driven Ad E1A expression or deletion of the Rb binding site in Ad E1A gene to achieve preferential replication in cancer cells (33–38). Most of these oAds also harbor a genetically engineered fiber on the viral capsid to enhance their cellular uptake into tumor cells in a coxsackie and adenovirus receptor (CAR)-independent manner: this is important in a clinical environment where heterogeneity of tumor can lead to variable or abrogated CAR expression levels that can lead to suboptimal infection by Ad with wild-type fiber (39, 40).

Indeed, phase I results of ICOVIR-5, an oAd that is being investigated in several ongoing clinical trials (NCT03714334, NCT03178032, NCT02798406), contains a functional copy of the E1B 55 kDa gene, and its cancer specificity is acquired through deletion in the Rb binding domain of the Ad E1A region and insertion of a tumor-targeting RGD motif at the fiber region of the viral capsid to improve its cancer specificity. Despite significant advancements made to the viral constructs, poor systemic administrability of the virus remain a major challenge within the field as an ideal cancer therapeutic should be systemically administrable to effectively treat noninjectable or metastatic lesions in advanced stages of cancer. Patients treated with ICOVIR-5 (single intravenous (IV) infusion of 1 × 10^11^ to 1 × 10^13^ viral particles (VP)) demonstrated that only a small portion of systemically administered virus could accumulate in melanoma metastases but ultimately failed to induce objective response (41). A phase I trial results of IV infused enadenotucirev also failed to induce clinically beneficial response in patients with epithelial solid tumors or those that underwent tumor resection (29). Progressive disease was observed in ~56% of patients treated with systemically administered enadenotucirev. Similarly, another phase I trial results of IV administered enadenotucirev in combination with paclitaxel in platinum-resistant ovarian cancer patients only led to overall response rate of 10% at the highest dose of the virus of 1 × 10^12^ VP, which was lower than those achieved by paclitaxel monotherapy in similar patient demographic (42). Collectively, these clinical findings demonstrate that the intravenous administration of oAd remain suboptimal in current state and majority of the ongoing clinical trials evaluating locoregional administration of oAds.

Although systemic delivery of oAds remain a major challenge, strategies to maximize the induction of systemic antitumor immune response mediated by oAds could be a more practical approach to maximize the antitumor effect of oAd in the noninjected lesions. Indeed, many of the ongoing or recruiting phase I trials (NCT01437280, NCT02143804, and NCT02365818) are evaluating oAds expressing pro-inflammatory cytokines (granulocyte-macrophage colony stimulating factor (GM-CSF), interleukin (IL)-2, IL-12) or co-stimulators (4-1BB or CD40 ligand (4-1BBL and CD40L) to enhance the induction of antitumor immune response mediated by these viruses (43, 44). Although clinical trial results for many of these oAd-expressing antitumor immune transgenes are not available, their increasing prevalence in the current clinical landscape strongly indicates that identifying the correct combination of therapeutic transgenes will be integral for maximizing oAd application in immmuno-oncology.

The therapeutic transgenes, which was initially and mainly applied to clinical studies, are IL-12 and GM-CSF. For example, Ad5-yCD/mutTKSR39rep-hIL12, an oAd expressing the human IL-12 gene and two suicide genes (yeast cytosine deaminase (yCD) and HSV thymidine kinase (TK), yielded promising antitumor immune response and tumor growth inhibition in pre-clinical and clinical studies (45–48). Both suicide genes, yCD (cytosine deaminase) and mutTKSR39 (HSV thymidine kinase), expressed by Ad5-yCD/mutTKSR39rep-hIL12 successfully converted respective prodrugs 5-fluorocytosine and ganciclovir to induce irreversible inhibition of DNA synthesis and yielded potent anti-tumor effects (49, 50). Further, Ad5-yCD/mutTKSR39rep-hIL12 treatment improved induction of antitumor immune response through expression of hIL12, as evidenced by activation of NKs and secretion of IFN-γ by cytotoxic T lymphocytes (CTL) against tumor cells (51). Based on these findings from the preclinical study, two phase-1 clinical trials (NCT02555397 and NCT03281382) have been initiated to evaluate Ad5-yCD/mutTKSR39rep-hIL12 for the treatment of patients with either prostate or metastatic pancreatic cancer, respectively (47).

There are two clinical trials ongoing with oAds-expressing GM-CSF (ONCOS-102 and CG0070). ONCOS-102, developed by Targovax, possesses a 24 bp deletion in the Rb binding site of E1A to improve its cancer specificity. ONCOS-102 has shown encouraging phase I results for patients heavily pretreated for solid tumors (38, 52). The patients treated with ONCOS-102 intratumoral injection of a dose range at 3 × 10^10^ VP, 1 × 10^11^ VP, or 3 × 10^11^ VP/injection on days 1, 4, 8, 15, 29, 57, 85, 113 and 141 showed stable disease in 40% of evaluable cases (of the 12 patients in this study, two passed away before the first clinical assessment). ONCOS-102 treatment elevated number of tumor-infiltrating cytotoxic CD8^+^ T cells and cancer-specific CD8^+^ T cells in blood, indicating systemic activation of the immune system. Importantly, activation of antitumor immune system correlated with overall survival. Furthermore, upregulation of programmed death ligand 1 (PD-L1) after treatment with ONCOS-102 suggested that the combination of ONCOS-102 with immune checkpoint inhibitors (ICI), including PD-1/PD-L1 axis inhibitors, offers a promising strategy to treat refractory tumors. In support, a clinical trial (NCT03003676) combining ONCOS-102 and Keytruda (pembrolizumab; an anti-PD-1 antibody) is under investigation.

Another GM-CSF-expressing oAd, CG0070, was assigned cancer specificity by transcribing Ad E1A through E2F-1 promoter. In a phase I trial, 10^12^ VP of CG0070 induced complete response (CR) in bladder cancer patients who did not respond to standard care (bacillus calmette-guerin (BCG) treatment). Recently, CG0070 showed success in a phase II study against BCG-unresponsive high-grade non-muscle invasive bladder cancer (NMIBC; NCT02365818). Recently, CG0070 completed phase II study in a successful manner against BCG-unresponsive high-grade non-muscle invasive bladder cancer (NMIBC; NCT02365818). In specific, it was reported in American Society of Clinical Oncology (ASCO) meeting that CR rate for CG0070-phase II trials in the single dose cohort was 23% (3/13) (53). Their findings showed that CR response rate was greatly improved in patients who received multiple injections of CG0070, reaching CR rate of 64% (14/22). Six patients from multiple dose cohorts remained in remission (duration ranging from 3.3 to 38.2 months) as of the last follow-up. Currently, phase III study of CG0700 monotherapy is ongoing (NCT04452591), while the combination of CG0070 with Keytruda is in phase II clinical trials for treatment of BCG-unresponsive NMIBC patients (NCT04387461). The clinical results of the combination therapy trial reported in April of 2022 reported that 89% of patients evaluable for efficacy (16/18) had CR at 3-month time point and 75% (8/18) maintained CR at the 12-month assessment (https://www.cgoncology.com/news/press-releases/041322/). Together, these reports demonstrate that arming oAds with immune stimulatory cytokines could improve overall patient response rate and clinical benefit.

LOAd703, which is under phase 1/2a clinical trial (NCT02705196 and NCT03225989), expresses trimerized CD40L and 4-1BBL as immune activators to stimulate the CD40 and 4-1BB pathways, respectively (34). Many cells in the tumor microenvironment, including stromal cells and the infiltrating immune cells, express CD40 and 4-1BB; thus, expression of complementary activating ligands *via* LOAd703 could activate many types of cells in the tumor milieu to induce antitumor immune response. For example, dendritic cells (DC) were stimulated by LOAd703 to upregulate co-stimulators, cytokines, and chemokines, ultimately leading to increased antigen-specific T cells and NK cell population to mount potent antitumor immune response. Interim phase I/II trial results reported in 2020 revealed that intratumoral administration of LOAd703 with nab-paclitaxel/gemcitabine chemotherapy was well-tolerated in pancreatic ductal adenocarcinoma patients (13 patients were evaluable); most adverse events were transient grade 1-2 with only a single patient at the highest dose (5 × 10^11^ VP) exhibiting dose-limiting grade 3 transaminase elevation. The decreased number of immunosuppressive myeloid-derived suppressor cells in circulation was reported (8/13), suggesting alleviation of immunosuppression. Further, elevated effector memory T cell (10/13) and tumor antigen-specific T cell counts were reported, ultimately leading to 6/10 patients at higher virus doses exhibiting partial response.

Collectively, there has been significant advancements in the genetic constructs of oAds that are currently being evaluated in clinical environment to enhance their safety profile and efficacy. Notably, there are increasing number of trials evaluating the oAds armed with pro-inflammatory immune transgenes to maximize the viruses’ potential to induce robust systemic antitumor immune response, which is an essential parameter to control the growth of noninjectable and metastatic lesions in patients with advanced stages of cancer.

### 2.2 Oncolytic herpes simplex virus

oHSV, like several other types of OVs, can directly kill tumor cells and promote antitumor immune response. The pathogenicity and function of viral proteins of HSV have been well-characterized, and most oHSVs in development have deletion of several viral genes to prevent potential neurotoxicity and confer cancer specificity (54). Furthermore, clinicians have proper knowledge, training, and means to treat HSV infection in an efficient manner, as HSV is one of the few viruses with well-established antiviral drugs. These attributes endow oHSV another extra layer of safety in the clinic since uncontrolled viremia and other virus-related adverse events can be managed by clinicians in an efficient manner. Additionally, nearly all types of cancer can be infected with oHSV, which is beneficial in clinical scenarios where heterogeneity of tumors and resulting phenotypic variations necessitate flexibility and wide target coverage to induce optimal therapeutic effect. oHSV also possesses a large genome size (55) and a relatively large transgene insertion capacity.

Talimogene laherparepvec, an oHSV expressing GM-CSF also known as Imlygic, was the first oncolytic virus to be approved by FDA and EMA. It was shown to possess promising antitumor activity in melanoma patients. This landmark approval led to significant improvement in understanding of OV mechanisms in patients, such as shedding, biodistribution, induction of antitumor immune response, and transmissibility. Imlygic usage and number of clinical reports have been on an upward trajectory, and its prevalence has improved clinicians’ and government regulators’ understanding and handling of oncolytic virotherapy in medical settings. The approval of Imlygic has accelerated the development of other OVs, and the promising clinical outcomes achieved *via* its combination with other clinically approved immunotherapeutics forecast their critical role in advancing cancer immunotherapy paradigm.

While Imlygic remains the only oncolytic HSV to be approved by US FDA and EMA to date, several other oHSVs are under clinical investigation (**Table 2**). Some of the earliest oHSV clinical trial results were published as early as 2002 (58), using HSV1716 that lacks γ_1_34.5. These findings showed that 1 × 10^5^ plaque-forming unit (PFU) of HSV1716 can be administered intratumorally in a safe manner without dose-limiting toxicity in both HSV-seropositive and -negative patients. HSV1716 replicated actively in brain tumors (in two of 12 patients, HSV genome copies were detected at a higher level than the input dose at 9 days after inoculation), and infectious particles were recovered from the tumor biopsies of these two patients. In another phase I trial, HSV1716 was injected into the normal brain tissues surrounding the resection cavity following surgical resection of glioma (59). Injection of HSV1716 into normal brain tissues did not induce any observable HSV1716-related toxicity. Three of the 12 enrolled patients remained alive and clinically stable at 15-22 months post-surgical resection and HSV1716 injection into normal brain tissues surrounding the resection cavity. Remarkably, one of the surviving patients who had extensive recurrent disease at the time of trial enrollment demonstrated reduction in residual tumor volume over the 22-month period after HSV1716 administration, despite not receiving any adjuvant treatment. The patient at this period remained in complete clinical and radiological remission. In 2017, a phase I trial of intratumorally-administered HSV1716 (a single dose of 10^5^ to 10^7^ PFU) in young cancer patients revealed that HSV1716 is safe and well-tolerated in young patients (60). However, there was no tumor shrinkage (either in injected or uninjected lesions) in any of these patients, suggesting further optimization of the clinical protocol will be necessary for future HSV1716 trials.

**Table 2:** Oncolytic HSVs tested in current clinical trials.

Oncolytic HSV	TumorSelectivity	Route of Administration	Combination	Indication	Phases	NCT Number	Sponsor/Collaborator	Status
**Talimogene Laherparepvec^1^** **(T-VEC, IMLYGIC)**	- γ_1_34.5 deletion -ICP 47 gene deletion	Intratumoral Injection	None	Cutaneous Squamous Cell Cancer	Phase 2	NCT03714828	-University of Arizona -Amgen	Recruiting
		Intratumoral Injection	Hypofractionated Radiotherapy	-Melanoma -Merkel Cell Carcinoma -Other Solid Tumors	Phase 2	NCT02819843	-Memorial Sloan Kettering Cancer Center -Amgen	Active, not recruiting
		Intratumoral Injection	Nivolumab	-Refractory Lymphomas -Advanced or Refractory Non-melanoma Skin Cancers	Phase 2	NCT02978625	-National Cancer Institute	Recruiting
		N/A	-Nivolumab - Trabectedin	Sarcoma	Phase 2	NCT03886311	-Sarcoma Oncology Research Center, LLC	Recruiting
		Intratumoral Injection	Pembrolizumab	Melanoma	Phase 2	NCT02965716	-National Cancer Institute	Active, not recruiting
		Intratumoral Injection	Pembrolizumab	Metastatic or advanced sarcoma	Phase 2	NCT03069378	-Memorial Sloan Kettering Cancer Center -Amgen -Merck Sharp & Dohme LLC	Recruiting
		Intratumoral Injection	None	Angiosarcoma of Skin	Phase 2	NCT03921073	H. Lee Moffitt Cancer Center and Research Institute	Active, not recruiting
		Intratumora Injection l	Nivolumab	Melanoma	Phase 2	NCT04330430	-The Netherlands Cancer Institute -Amgen	Recruiting
		Intratumoral Injection	Radiation	Soft tissue sarcoma	Phase 2	NCT02923778	-National Cancer Institute	Recruiting
		Intratumoral Injection	None	Melanoma	Phase 2	NCT04427306	-University of California, Davis -Amgen	Recruiting
		Intratumoral Injection	Pembrolizumab	Cutaneous Melanoma	Phase 2	NCT03842943	University of Louisville	Recruiting
		Intratumoral Injection	Pembrolizumab	Anti-PD-1 therapy refractory melanoma	Phase 2	NCT04068181	-Amgen -Merck Sharp & Dohme LLC	Active, not recruiting
		Intratumoral Injection	None	Kaposi Sarcoma	Phase 2	NCT04065152	Assistance Publique - Hôpitaux de Paris	Recruiting
**TBI-1401** **(HF10, Canerpaturev, C-REV)**	Unknown^2^	Intratumoral Injection	None	Solid Tumor	Phase 1	NCT02428036	Takara Bio Inc.	Completed
		Intratumoral Injection	None	Refractory Head and Neck Cancer, Solid Tumors With Cutaneous and/or Superficial Lesions	Phase 1	NCT01017185	Takara Bio Inc.	Completed
		Intratumoral Injection	Gemcitabine, Nab-paclitaxel, TS-1	Unresectable Pancreatic Cancer	Phase 1	NCT03252808	Takara Bio Inc.	Active, not recruiting
		Intratumoral Injection	Ipilimumab	Unresectable or Metastatic Melanoma	Phase 2	NCT03153085	- Takara Bio Inc.	Completed
		Intratumoral Injection	Ipilimumab	Malignant Melanoma	Phase 2	NCT02272855	-Takara Bio Inc. -Theradex	Completed
		Intratumoral Injection	Nivolumab	Resectable Melanoma	Phase 2	NCT03259425	- Takara Bio Inc. -Bristol-Myers Squibb -University of Utah	Terminated
**HSV1716**	- γ_1_34.5 deletion	Intreapleural Injection	none	Malignant Pleural Mesothelioma	Phase 1/2	NCT01721018	Virttu Biologics Limited	Completed
		Intratumoral Injection or Intravenous Injection	none	Non-CNS Solid Tumors	Phase 1	NCT00931931	-Timothy Cripe -Nationwide Children's Hospital	Completed
		Injection into resection cavity	dexamethasone	Refractory or Recurrent High Grade Glioma	Phase 1	NCT02031965	-Pediatric Brain Tumor Consortium -National Cancer Institute (NCI)	Terminated
**G207**	- γ_1_34.5 deletion -U_L_39 substitution with LacZ gene	Intratumoral & tumor bed at the resection site	None	Recurrent Brain Cancer	Phase 1/2	NCT00028158	MediGene	Completed
		Intratumoral Injection	None	Recurrent or Refractory Cerebellar Brain Tumors in Children	Phase 1	NCT03911388	University of Alabama at Birmingham	Recruiting
		Intratumoral Injection	Radiation	Progressive or Recurrent Supratentorial Brain Tumors in Children	Phase 1	NCT02457845	University of Alabama at Birmingham	Recruiting
		Intratumoral Injection	Radiation	Glioma	Phase 1	NCT00157703	- MediGene --National Cancer Institute	Completed
		Intratumoral Injection	Radiation	Recurrent glioma in children	Phase 2	NCT04482933	- University of Alabama at Birmingham - Treovir, LLC	Not yet recruiting
**NV1020**	-deletion of a 15-kb region at the U_L_/_S_ junction containing one copy of the α0, α4, and γ_1_34.5 -deletion to one copy of U_L_56 gene	Intrahepatic arterial injection	None	Colorectal cancer metastatic to the liver	Phase 1	NCT00012155	-Memorial Sloan Kettering Cancer Center -National Cancer Institute	Completed
		Intrahepatic arterial injection	None	Colorectal cancer metastatic to the liver	Phase 1/2	NCT00149396	MediGene	Completed
**OH2** **(rHSV2hGM-CSF)**	-Mutation of ICP6 gene - γ_1_34.5 deletion	Intratumoral Injection	-irinotecan, -HX008 (PD-1 ICI)	Solid Tumor	Phase 1/2	NCT03866525	Wuhan Binhui Biotechnology	Recruiting
		Intravesical Injection	None	Non-muscle-invasive Bladder Cancer	Phase 1/2	NCT05232136	Wuhan Binhui Biotechnology	Recruiting
		Intravesical Injection	None	Pancreatic Cancer	Phase 1/2	NCT04637698	Wuhan Binhui Biotechnology	Recruiting
		Intratumoral Injection	None	Central Nervous System Tumors	Phase 1/2	NCT05235074	Wuhan Binhui Biotechnology	Recruiting
		N/A	pembrolizumab	Solid Tumor		NCT04386967	Wuhan Binhui Biotechnology	Recruiting
		Intratumoral Injection	None	Advanced Bladder Carcinoma	Phase 2	NCT05248789	Wuhan Binhui Biotechnology	Not yet recruiting
		Intratumoral Injection	-LP002 (PD-L1 ICI) -Cisplatin -Fluorouracil	Cancer of digestive track	Phase 1	NCT04755543	Taizhou HoudeAoke Biomedical Co., Ltd.	Recruiting
		N/A	-HX008 (PD-1 ICI)	Melanoma	Phase 1/2	NCT04616443	Wuhan Binhui Biotechnology	Recruiting
		Intratumoral Injection	-HX008 (PD-1 ICI) -Radiation	Melanoma with liver metastasis	Phase 1	NCT05068453	Beijing Cancer Hospital	Not yet recruiting
		Intratumoral Injection	-HX008 (PD-1 ICI) -Axitinib	Melanoma with liver metastasis	Phase 1	NCT05070221	Beijing Cancer Hospital	Not yet recruiting
**rQNestin34.5v.2**	- γ_1_34.5 deletion -U_L_39 deletion -Nestin promoter driven expression of single γ_1_34.5 gene	N/A	Cyclophosphamide	Recurrent Malignant Glioma	Phase 1	NCT03152318	-Dana-Farber Cancer Institute -National Institutes of Health -Candel Therapeutics, Inc.	Recruiting
**C134**	- γ_1_34.5 deletion -Expression of human cytomegalovirus protein kinase R evasion protein IRS1	Intratumoral Injection	none	Recurrent glioblastoma	Phase 1	NCT03657576	-University of Alabama -National Cancer Institute	Recruiting
**M032** **(NSC 733972)**	Substitution of γ_1_34.5 and ORF P gene with α27-*tk*	Intratumoral Injection	none	Brain Cancer	Phase 1	NCT02062827	University of Alabama at Birmingham	Active, not recruiting
		Intratumoral Injection	Pembrolizumab	Brain Cancer	Phase 1/2	NCT05084430		Active, not recruiting
**OrienX010**	Not available	Intratumoral Injection	none	Melanoma, Liver, Pancreatic, and Lung Cancer	Phase 1	NCT01935453	-OrienGene Biotechnology -START Shanghai -Beijing Bozhiyin T&S Co., Ltd.	Completed
		Intratumoral Injection	JS001 (PD-1 ICI)	Melanoma	Phase 1	NCT04206358	Beijing Cancer Hospital	Recruiting
		Intratumoral Injection	Toripalimab (PD-1 ICI)	Melanoma	Phase 1	NCT04197882	Beijing Cancer Hospital	Active, not recruiting
		Intratumoral Injection	Dacarbazine	Melanoma	Phase 2	NCT04200040	OrienGene Biotechnology	Recruiting
**RP1**	- γ_1_34.5 deletion -ICP 47 gene deletion	Intratumoral Injection	None	Advanced cutaneous cancer	Phase 1/2	NCT04349436	Replimune Inc	Recruiting
		Intratumoral Injection	Nivolumab	Solid tumors	Phase 2	NCT03767348	Replimune Inc	Recruiting
		Intratumoral Injection	Cemiplimab	Advanced squamous skin cancer	Phase 2	NCT04050436	-Replimune Inc -Regeneron Pharmaceuticals	Recruiting
**RP2**		Intratumoral Injection	Nivolumab	Solid tumors	Phase 1	NCT04336241	Replimune Inc	Recruiting
**RP3**		Intratumoral Injection	Nivolumab	Solid tumors	Phase 1	NCT04735978	-Replimune Inc -Bristol-Myers Squibb	Recruiting
**VG161**	- γ_1_34.5 deletion	Intratumoral Injection	None	Liver cancer	Phase 1	NCT04806464	CNBG-Virogin Biotech (Shanghai) Ltd.	Recruiting
		Intratumoral Injection	None	Solid Tumor	Phase 1	NCT04758897	CNBG-Virogin Biotech (Shanghai) Ltd	Recruiting
		Intratumoral Injection	Nivolumab	Advanced pancreatic cancer	Phase 1/2	NCT05162118	Zhejiang University	Recruiting
		Intratumoral Injection	None	Hepatocellular Carcinoma or Intrahepatic Cholangiocarcinoma	Phase 2	NCT05223816	-Virogin Biotech Canada Ltd -Virogin Biotech Ltd.	Not yet recruiting
**VG2025**	-Tumor-specific CEA promoter driven ICP27 gene expression	Intratumoral Injection	None	Solid Tumor	Phase 1	NCT05266612	-Virogin Biotech Canada Ltd -Virogin Biotech Ltd	Not yet recruiting
**rRp450**	-replacement of U_L_39 gene encoding ICP6 with rat Cyp2b1 gene	Hepatic artery infusion	None	Liver metastases and liver cancer	Phase 1	NCT01071941		Recruiting
**T3011** **(MVR-T3011)**	Unknown	Intravenous	Pembrolizumab	Advanced or metastatic solid tumors	Phase 1/2	NCT04780217	ImmVira Pharma Co. Ltd	Recruiting
**T3011** **(MVR-T3011)** **C5252** **(MVR-C5252)**	Unknown	Intratumoral	Pembrolizumab	Advanced or metastatic solid tumors	Phase 1/2	NCT04370587	ImmVira Pharma Co. Ltd	Recruiting
		Intratumoral	None	Recurrent or progressive glioblastoma	Phase 1	NCT05095441	ImmVira Pharma Co. Ltd	Recruiting

1. Only “Active” or “Recruiting” phase II or higher clinical trials using talimogene laherparepvec have been listed in the table due to the largest number of clinical trials being performed to date for a single OV product. 2. HF10 does not express several UL genes, but retains g134.5. Precise mechanism of HF10’s cancer specificity has not been elucidated at present. Detailed discussion of mutations in HF10 gene can be found in REFS (56, 57).

In a phase I glioma clinical trial using G207 (with deletion of γ_1_34.5 and inactivated U_L_39), the virus was intratumorally administered pre-resection than subsequently into the normal brain tissues surrounding the resection cavity post-resection of recurrent glioblastoma multiforme (GBM) (61). In detail, six patients were treated initially with 1.5 × 10^8^ PFU *via* stereotactic injection into the GBM tumor (pre-resection), followed by tumor resection at 2 to 5 days after the final virus administration. Immediately after surgical resection, second dose of G207 was administered into resected tumor bed using multiple injections. The viral replication was noted in resected tumor tissues in 50% of the patients. Although no determination regarding efficacy could be made due to the small cohort, the injected tumor lesions showed elevated T cell, monocyte, and macrophage infiltration in comparison to those observed prior to G207 administration, which would be integral to induction of OV-mediated antitumor immune response. G47Δ, a third-generation oHSV based on G207, harbors addition deletion of the α47 gene and has been under extensive clinical investigation in Japan (62). A phase I-IIa clinical trial of G47Δ in patients with recurrent glioblastoma was completed in 2014 (UMIN000002661), and a subsequent phase II trial examining (UMIN000015995) multiple stereotactic administration at 1 × 10^9^ PFU (a maximum of six times) revealed that locally administered G47Δ was well tolerated. In February 2016, G47Δ was designated as a breakthrough therapy drug by the Ministry of Health, Labor, and Welfare of Japan (63) and it has been given conditional approval for the treatment of patients with malignant glioma or any primary brain cancer in 2021 (64).

Imlygic was the only antitumor cytokine-expressing oncolytic HSV being evaluated in phase II/III clinical trials as of 2019, with other oHSVs in phase II/III trials not expressing any therapeutic transgenes, thus failing to fully exploit the large transgene capacity of oHSV. With growing number of preclinical data demonstrating that oHSVs expressing any antitumor immune transgene exert more potent tumor growth inhibition than do cognate controls lacking any transgenes (65–68), majority of the recruiting or ongoing clinical trials (14 out of 17) listed on http://clinicaltrials.gov as of July 2022 utilizes an oHSV expressing at least one antitumor immune transgene. Currently, combinations of various immune stimulatory transgenes are being actively explored in either preclinical stage or early phase of clinical trial to improve antitumor immunogenicity of oHSVs. Notably, in view of the reports suggesting that the effect of potent antitumor cytokines like IL-12 supersedes the tumor growth inhibiting effect of GM-CSF, the transgene payload of oHSV is diverging away from GM-CSF of Imlygic (69). In lieu of these trends, there are increasing number of oHSV pipelines harboring IL-12 as a transgene in clinical trials: two phase I clinical trial utilizing an oHSV expressing IL-12 (M032) against recurrent malignant glioma (NCT02062827 and NCT02062827) (70), three phase I or phase I/II trials examining either intratumorally or IV administered C5252 or MVR-T3011 (both are oHSV co-expressing IL-12 and PD-1 antibody in NCT04370587, NCT04780217, and NCT05095441), three phase I or phase I/II trials evaluating VG161 (oHSV co-expressing IL-12, IL-15 with its receptor α unit, and Fc-fused PD-L1 blocking peptide in NCT04806464, NCT05162118, and NCT04758897), and a single phase I study of ONCR-117 (an oHSV expressing IL-12, extracellular domain of FLT3LG, CCL4, anti-CTLA-4 ICI ipilimumab, and anti-PD-1 single variable heavy chain domain fused with Fc region NCT04348916) is ongoing.

### 2.3 Oncolytic vaccinia virus

Vaccinia virus (VV) is a membrane-coated virus with a linear double-strand DNA virus and was shown to efficiently infect, replicate, and kill a wide-range of cancer cells (71). Further, large viral genome size of VV allows insertion of large transgenes (~40 kb) with minimal change in viral production (72). VV also possesses attributes that ensure good safety profile: (1) VV replication cycle occurs in cytoplasm (73), thus there is no risk of genome integration (74) and (2) there is no associated-human disease (72) reported so far. However, one major shortcoming is that 50% of VV genes have unknown functions, which can lead to unforeseeable side-effects when interacting with other cancer therapeutics (72). Despite incomplete understanding of VV viral proteins, development of various oncolytic VV (oVV)s has been pursued since the 1990s (75–77).

The most extensively tested oVV in clinical trials is Pexa-Vec (pexastimogene devacirepvec, also known as JX-594) that expresses human GM-CSF (78). Pexa-Vec was well-tolerated in patients with refractory solid tumors, showing a good safety profile (NCT01169584) (79). Importantly, a phase II clinical trial of Pexa-Vec in combination with sorafenib was shown to improve long-term survival rate (~35% and 11% at 18 months for respectively high-dose and low-dose groups of virus injection) of liver cancer patients (NCT00554372) (80). Other promising clinical results demonstrated that Pexa-Vec treatment elevated IFN-γ and tumor necrosis factor (TNF)-α in tumor sites to lead to activation and/or recruitment of neutrophils, eosinophils, and lymphocytes to the tumor tissues, ultimately suggesting immune activation at injected tissues (79–82). Furthermore, tumor vascular disruption was also observed (80), suggesting an anti-vascular effect of Pexa-Vec. Despite these results from phase I/II clinical trials, Independent Data Monitoring Committee recently concluded that phase III trial results of Pexa-Vec in combination with sorafenib for liver cancer (NCT02562755) failed to improve the clinical outcome of patients in respect to the standard care.

Although phase III trial results of Pexa-Vec have been disappointing, there are several other oVVs in clinical trials targeting wide range of tumor types that might yield promising results (**Table 3**). For example, phase I trial results of oVV (vvDD) derived from Western Reserve strain, which is the most virulent strain of VV, has shown some promising outcomes (83). Two viral genes (TK and vaccinia growth factor (VGF) genes) have been deleted in vvDD to endow tumor specificity and decrease viral replication in resting cells (84). Subsequently, vvDD was engineered to co-express somatostatin receptor (SR) to track the virus easily in an *in vivo* setting (85) and cytosine deaminase (CD) as a suicide gene (86), generating a vvDD-CDSR (also known as JX-929) that entered phase I clinical trial. In 2015, phase I trial results of intratumorally administered JX-929 demonstrated good safety profile and tumor specificity (83). In specific, infectious JX-929 particle was detected in the injected lesions in 4 of 5 biopsied patients in a high-dose cohort (1 x 10^8^ to 3 x 10^9^ PFU), whereas 3/3 biopsy samples from injected lesions in a low-dose cohort (3 x 10^7^ PFU) were negative for infectious particles at 8-day post administration. Notably, 4 patients exhibited infectious viral persistence in the injected lesion, and 50% of these patients tested positive for infectious JX-929 in the non-injected lesions, suggesting distal viral spread from the injected site. Antitumor activity and tumor regression were observed in injected lesions for 2 of 3 patients with active viral replication in higher dose cohorts, but numerous other non-injected nodules failed to show any sign of infection or respond to treatment. CD4^+^ and CD8^+^ T cell populations among peripheral blood mononuclear cells (PBMC)s of 1 ×10^9^ and 3 × 10^9^ PFU dose cohorts showed dose-dependent increase in the levels of pERK, pS6, and Ki67, suggesting T cell proliferation. JX-929 did not induce any significant elevation in serum chemokine or cytokine levels. Interestingly, one patient with a large tumor burden who had received two injections of JX-929 under a compassionate use protocol showed complete resolution of both injected tumors, thus demonstrating a potentially promising antitumor effect of JX-929.

**Table 3:** Oncolytic vaccinia viruses tested in current clinical trials.

Oncolytic vaccinia virus	Tumor Selectivity	Route of Administration	Combination	Indication	Phases	NCT Number	Sponsor/Collaborators	Status
**GL-ONC1**	generating interruptions in the thymidine kinase, F14.5L and hemagglutinin genes.	Intraperitoneal injection	Bevacizumab	Recurrent or Refractory Ovarian Cancer	Phase 1, Phase 2	NCT02759588	Genelux Corporation	Recruiting
		Intraperitoneal Injection	none	Advanced Peritoneal Carcinomatosis	Phase 1|Phase 2	NCT01443260		Completed
		Intravenous Injection	none	Advanced Solid Tumors	Phase 1	NCT00794131		Completed
		Intravenous Injection	Cisplatin, Radiotherapy	Head & Neck Cancer	Phase 1	NCT01584284		Completed
		Intravenous Injection	Surgery	Solid Organ Cancers Undergoing Surgery	Phase 1	NCT02714374	Kaitlyn Kell intravenousy, MD,Genelux Corporation, University of California, San Diego	Active, not recruiting
**Pexa-Vec** **(JX-594)**	Thymidine Kinase-Deactivated Vaccinia Virus Plus GM-CSF	Intravenous Injection	none	Peritoneal Carcinomatosis of Ovarian Cancer Origin	Phase 2	NCT02017678	Andrea McCart, Ontario Institute for Cancer Research, Mount Sinai Hospital, Canada	Withdrawn
		Intratumoral Injection	none	Liver Cancer (Failed Sorafenib)	Phase 2	NCT01387555	Jennerex Biotherapeutics	Completed
		Intratumoral Injection	none	Primary or Metastatic Hepatic Carcinoma	Phase 1	NCT00629759	Jennerex Biotherapeutics, Green Cross Corporation	Completed
		Intravenous Injection	none	Refractory Solid Tumors	Phase 1	NCT00625456	Jennerex Biotherapeutics, SillaJen, Inc.	Completed
		Intratumoral Injection	none	Refractory Solid Tumors in Pediatric Patients	Phase 1	NCT01169584	Jennerex Biotherapeutics, SillaJen, Inc.	Completed
		Intravenous Injection	Irinotecan	Metastatic, Refractory Colorectal Carcinoma	Phase 1, Phase 2	NCT01394939	Jennerex Biotherapeutics, Transgene, SillaJen, Inc.	Completed
		Intratumoral Injection	none	Malignant Melanoma	Phase 1, Phase 2	NCT00429312	Jennerex Biotherapeutics	Completed
		Intratumoral Injection	none	Unresectable Primary Hepatocellular Carcinoma	Phase 2	NCT00554372	Jennerex Biotherapeutics, SillaJen, Inc.	Completed
		Intravenous Injection	Durvalumab	Refractory Colorectal Cancer	Phase 1/2	NCT03206073	National Cancer Institute (NCI), National Institutes of Health Clinical Center (CC)	Recruiting
		Intratumoral Injection	Ipilimumab	Metastatic / Advanced Solid Tumors	Phase 1	NCT02977156	Centre Leon Berard|Transgene	Recruiting
		Intravenous Injection/ Intratumoral Injection	REGN2810 (anti-PD-1)	Renal Cell Carcinoma	Phase 1	NCT03294083	SillaJen, Inc., Regeneron Pharmaceuticals	Recruiting
			Sorafenib	Hepatocellular Carcinoma	Phase 3	NCT02562755	SillaJen, Inc.	Active, not recruiting
**ASP9801**	Deletion of VGF and O1L gene	Intratumoral Injection	none	Advanced Metastatic Solid Tumors	Phase 1	NCT03954067	-Astellas Pharma Global Development, Inc., -Astellas Pharma Inc	Recruiting
**TBio-6517** **(TAK-605)**	Unknown	Intratumoral Injection	Pembrolizumab	Solid tumors	Phase 1/2	NCT04301011		Recruiting
**RGV004**	Deletion to thymidine kinase gene	Intratumoral Injection	None	Refractory/Relapsed B-cell Lymphoma	Phase 1	NCT04887025	-Second Affiliated Hospital, School of Medicine, Zhejiang University -Hangzhou Rongu Biotechnology Co., Ltd.	Not yet recruiting
**T601**	-Deletion to thymidine kinase gene -Deletion to Ribonucleotide Reductase gene	N/A	5-FC	Solid tumors	Phase 1/2	NCT04226066	Tasly Tianjin Biopharmaceutical Co., Ltd.	Recruiting
**TG6002** **(VV TK-RR-FCU1)**	-Deletion to thymidine kinase gene -Deletion to Ribonucleotide Reductase gene	Intrahepatic arterial administration	5-FC	Metastatic colorectal cancer	Phase 1/2	NCT04194034	Transgene	Recruiting
		Intravenous injection	5-FC	Advanced gastrointestinal tumor	Phase 1/2	NCT03724071	Transgene	Recruiting

Another phase I trial of JX-929 where IV administration was employed against solid tumors (NCT00574977) demonstrated a good safety profile with no dose-limiting toxicity or serious adverse events in cohorts ranging from 3 × 10^8^ to 3 × 10^9^ PFU (87). At 4 h post systemic administration, significant elevation of Th1 or Th1-related cytokines (IL-2, IFN-γ, IL-7, and GM-CSF) was observed, while the expression level of Th2 cytokines remained unaffected after the treatment, suggesting acute Th1 immune activation likely due to antiviral immune response. Most patients were cleared of the virus quickly after IV infusion, and the viral genome was only detected in tumor biopsies of 2 patients on days 8 and 22 after treatment, demonstrating that systemic administration of JX-929 leads to insufficient viral accumulation in tumor tissues. Poor viral accumulation in tumor tissues likely resulted in the poor antitumor activity of IV-administered JX-929, as evidenced by failure to show any sign of necrosis or change in PET signal intensity on PET-CT scan results at 3 weeks post-administration.

There are other clinical cases using different oVVs clearly illustrating that systemic administration of oVV should be avoided in future clinical trials. Two phase I trials evaluating two systemic administration routes (IV and intraperitoneal injection - NCT01584284 and NCT01443260)) for Lister strain oVV (also known as GL-ONC1 or GLV-1h68) failed to elicit an antitumor effect (88, 89). In detail, a phase I trial evaluating IV-administered GL-ONC1 in combination with chemoradiotherapy to treat head and neck cancer stage IV patients showed that IV infusion was safe with no grade 4 toxicity observed and 18 of 19 patients completed the injection course. Of the 14 patients bearing p16-negative tumors, 7 deaths and 7 treatments failures were observed by 30 months. Despite the lack of virus accumulation in tumor tissues and the absence of significant improvement, the combination therapy (NCT01584284) is safe and that the viral MTD was not reached (88). Similarly, intraperitoneal infusion of GL-ONC1 in late stage carcinomatosis patients failed to exert a meaningful antitumor effect. Despite the increase in lymphocyte count in the peritoneal cavity and even though 8 of 9 patients showed efficient infection in ascitic fluids, only 4 of them had virus-infected cells in peritoneal fluids. As a consequence, of the 4 patients who completed the 4-cycle-treatment, only 2 had stable disease. Furthermore, grade 4 adverse events were not observed at any dosage. However, no correlation could be made with the virus dosage levels.

Collectively, these clinical trial results of oVV therapy clearly demonstrated that intratumoral injection of oVV should be the preferred route of administration in future clinical trials as systemic administration cannot sufficiently deliver oVV to tumor tissues to induce notable therapeutic effect. Furthermore, the limitation of the oVV therapy might be due to the highly advanced stage of patients used in clinical trials, but absence of critical side-effects is a big advantage of the vaccinia virus. The clinical benefits of oVV may be enhanced further with combination therapy such as radio-, chemo- or immunotherapy, as discussed in Section 3 of the review.

### 2.4 Other OVs

Although oAd, oHSV, and oVV have been most extensively evaluated in clinical environment (90) and primary scope of the review, there are other types of viruses, like reovirus, measles virus (MV), and picornaviruses, that are currently being evaluated in the clinical environment. Among these OVs, reovirus has been most extensively evaluated in clinical environment across multiple phase I and II clinical trials across multiple types of tumors, and thus will be discussed in-depth. Some of the other OVs with recently completed clinical studies and ongoing clinical trials will also be highlighted in this section of the review (**Table 4**).

**Table 4:** Characteristics of oncolytic viruses.

	DNA			RNA		
	Adenovirus	Herpesvirus	Vaccinia virus	Measles virus	Reovirus	Picornavirus
**Genome structure and size**	dsDNA 26-45 kb	dsDNA 120-240 kb	dsDNA 130-280 kb	ss (-) RNA 15.2-15.9 kb	dsRNA 18.2 - 30.5 kb	ss (+) RNA 7-8 kb
**Virion Capsid** **Symmetry**	Naked Icosahedral	Enveloped Icosahedral	Enveloped Complex	Enveloped Helical	Naked Icosahedral	Naked Icosahedral
**Transgene** **capacity**	**++**	**+++**	**+++**	**+**	**+**	**+**
***In vivo* gene expression**	**+++**	**+++**	**+++**	**++**	**++**	**++**
**Immunogenicity**	**+++**	**+++**	**++**	**++**	**++**	**+**
**Stability**	**++**	**++**	**++**	**+**	**+**	**+**
**Selectivity**	**+++**	**++**	**++**	**++**	**++**	**++**
**Safety**	**+++**	**++**	**++**	**++**	**++**	**++**
**Viral yield**	**+++**	**+**	**++**	**+++**	**+**	**+**
**Systemic delivery**	**-**	**-**	**+**	**+**	**+**	**+**

Reference (91–93).-, very low; +, low; ++, intermediate; +++, high.

#### 2.4.1 Oncolytic reovirus

A mammalian orthoreovirus type three Dearing strain, previously known as Reolysin and now manufactured as pelareorep, is one of the most extensively evaluated OV in clinical trials. Pelareorep is a non-enveloped and double-stranded RNA virus that is known to be relatively nonpathogenic in adults. The first-in-man phase I study of pelareorep, REO-001, enrolled 19 patients with accessible and advanced solid tumors that were intratumorally injected with the virus (94). No dose limiting toxicities were observed and majority of the treatment-related adverse effect being grade two or below, and tumor responses were observed in 37% of the patients. Subsequent phase I clinical trials investigating systemically administered pelareorep demonstrated that IV administered virus was well-tolerated in patients (36, 95, 96). Despite its safety, IV administered pelareorep as monotherapy only elicited modest therapeutic benefit across multiple trials (97).

Due to inconsistent and insufficient therapeutic benefit of pelareorep in multiple clinical trials as monotherapy, a series of phase II trials were launched to evaluate IV administered pelareorep in combination with standard of care chemotherapy across different types of cancer (1: pancreatic adenocarcinoma, 2: recurrent ovarian, tubal, or peritoneal cancer, 3: metastatic non-small cell lung cancer, 4: metastatic colon cancer, 5: advanced melanoma, and 6: metastatic breast cancer) and the results from these trials were published during 2016 to 2018 (98–102). Unfortunately, majority of these trials (4 out of 6) demonstrated that pelareorep in combination with standard of care chemotherapy failed to improve progression-free survival period compared with chemotherapy alone (98–101); 3 out of 4 trials also reported increased risk of severe adverse events (grade 3 or 4) in the pelareorep combination arm versus chemotherapy arm. Still, two of these phase II trials yielded potentially promising results when pelareorep was used in combination with standard of care chemotherapy for the treatment of patients with advanced melanoma or metastatic breast cancer (98, 103). In detail, pelareorep in combination with carboplatin and paclitaxel in patients with advanced melanoma met the efficacy goal for the first stage of the trial design with partial responses being observed in 3 out of 14 patients (ORR of 21%), stable disease in 9 out of 14 patients, median PFS of 5.2 months, and OS of 10.9 months (98); median PFS and OS showed minor improvement compared with historical controls (5.2 vs. 3 months & 10.9 vs. 9 months, respectively). Despite meeting the efficacy goal in the first stage of the trial, the second stage was terminated due to success of novel targeted therapies and immunotherapy for the treatment of melanoma during the course of first stage of this trial. The multicenter and randomized phase II trial that enrolled 74 patients with previously treated metastatic breast cancer demonstrated that combination of pelareorep with paclitaxel significantly improved the median OS versus paclitaxel alone (17.4 vs. 10 months, respectively), despite no differences being observed in median PFS and disease response rate between the two arms. Despite this substantial difference in OS, the result should be interpreted with caution as the study was not powered to detect a difference in OS and the study cohort favored pelareorep combination arm as the OS for the control chemotherapy arm was lower than expected.

As the combination of pelareorep with standard of care chemotherapy was largely unsuccessful across multiple types of cancer in several clinical trials, more recent clinical development using pelareorep has focused on the immune stimulatory aspect of pelareorep and are being conducted in combination with ICIs (NCT04102618, NCT04215146, NCT04445844, NCT03723915, NCT03605719, and Eudra-CT Number: 2020-003996-16) or GM-CSF (NCT02444546) with only two of the ongoing trials being evaluated in absence of other cancer immunotherapeutics for the treatment of patients with relapsed or refractory myeloma (NCT02101944 and NCT02514382). Two recent clinical studies demonstrated that this strategy of combining pelareorep with other cancer immunotherapy may yield promising results (104). In detail, PBMC isolated from metastatic colorectal cancer patients treated with pelareorep and chemotherapy in a phase I trial revealed that several pro-inflammatory cytokines IL-12p40, IL-12p70, GM-CSF, and IFN-γ were upregulated at day 8 or 15 after IV administration of pelareorep compared to the baseline, and reduction of pro-tumoral chemokines associated with angiogenesis or immunosuppression, like IL-8, VEGF, and RANTES/CCL5, was observed. Ultimately, pelareorep infusion induced APC stimulation and activation of T cells, suggesting that pelareorep could initiate antitumor and pro-inflammatory immune response (105).

Similarly, a phase Ib study evaluating the combination of pelareorep, PD-1-targeted ICI pembrolizumab, and standard chemotherapy for the treatment of patients with advanced pancreatic adenocarcinoma revealed that the combination therapy increased number of CD8^+^ T cells in tumor tissues in 2 out of 7 evaluable patients. Pelareorep infusion prior to pembrolizumab administration was shown to elevate the expression level of CTL attracting cytokines CXCL10 and CXCL11 in the peripheral blood of patients as well as promoting clonal expansion of T cells; the effects were further augmented upon additional treatment with pembrolizumab in patients. The study demonstrated that increased clonal expansion of T cells in patients positively correlated with higher OS, as patient who achieved partial response for 17.4 months and two patients who achieved stable disease for 4 and 9 months all exhibited higher peripheral T cell clonality, which is indicative of increased generation of tumor-associated neoantigens (106), as well as elevated expression level of antitumor cytokines, suggesting that pelareorep may inflame the tumor microenvironment and improve the efficacy of concomitantly administered ICI treatment. Based on these preliminary findings, a phase II trial evaluating the combination of pelareorep with pembrolizumab for the treatment of pancreatic cancer patients was initiated in 2018 (NCT03723915). Unfortunately, the combination therapy failed to meet the stage 1 evaluation criteria, which was to reach two or more PR or CR in patients from stage 1, thus ultimately leading to termination of the trial.

Despite the early termination of phase II trial exploring pelareorep and pembrolizumab, the interim results from phase I/II trial evaluating the combination of pelareorep and anti-PD-L1 ICI atezolizumab (Eudra-CT Number: 2020-003996-16) for the treatment of advanced gastrointestinal cancers revealed that the combination therapy administered to 3 out of 3 patients with locally advanced/metastatic unresectable pancreatic ductal adenocarcinoma led to partial response at week 16 after the treatment with no safety signals. Similarly, interim results from the phase I trial evaluating the combination of pelareorep and atezolizumab in patients with early breast cancer (NCT04102618) that are hormone receptor-positive and HER2-negative were promising (107). The study evaluated CelTIL score, a metric that quantitates changes in tumor cellularity and TIL with higher score correlating to favorable therapeutic responses, and met the primary endpoint of the trial when greater than 30% increase in CelTIL score was achieved in 40% of the patients receiving pelareorep in absence of atezolizumab and 60% in the combination therapy arm. Their findings demonstrated that increased CelTIL score was associated with (1) upregulation of PD-L1 expression level and (2) higher infiltration of CD8^+^ or memory T cells in the tumor tissues, as well as higher CD8^+^ T cell to Treg ratio, which are all indicative of polarization toward pro-inflammatory response and amelioration of tumor-induced immunosuppression. A gene panel analysis comparing the biopsy samples from pre-treatment and day 21 after the treatment revealed that aggressive luminal B breast cancer subtype was converted to luminal A subtype (with 100% conversion being achieved in the combination therapy arm) and panel of risk factors associated with tumor recurrence was markedly decreased in both pelareorep alone and pelareorep plus atezolizumab arms.

Collectively, IV infusion of pelareorep has been shown to be well-tolerated and induce pro-inflammatory changes to the tumor microenvironment across multiple types of cancers in different trials, but the therapeutic efficacy of the agent as monotherapy or in combination with standard of care chemotherapy were largely underwhelming. Although more recent clinical development strategy centered on immune stimulatory property of pelareorep seems to be yielding promising results as demonstrated by the interim results of two trials examining pelareorep in combination with anti-PD-L1 ICI atezolizumab, these initial findings should be taken with caution as the promising results of phase I trial exploring the combination of pelareorep with pembrolizumab in pancreatic cancer patients did not translate to successful phase II trial.

#### 2.4.2 Oncolytic measles virus

Measles virus (MV) is an enveloped RNA virus with a long history of antitumor activity in lymphoma patients, as there were many case studies from the 1970~80s reporting tumor regression or “spontaneous” remission following infection with MV (108). Due to this historical background, first in-human clinical trial of Edmonston vaccine strain of MV was conducted in patients with cutaneous T-cell lymphomas (109). The live-attenuated Edmonston vaccine strain of MV has a long history of excellent safety record, as it has been administered to vaccinate countless children, and it predominantly internalizes into the cells *via* CD46, which is known to be overexpressed in many cases of human tumors (109–112). In support, the CD46 was either shown to be expressed in tumor tissues or at a higher level in malignant tissues than the normal counterpart across multiple clinical studies evaluating oncolytic MV: the tumor biopsies from 5 out of 5 patients with cutaneous T-cell lymphomas tested positive for CD46 (109), 13 out of 15 patients with ovarian cancer showed high expression level of CD46 (110), and CD138^+^ myeloma cells from patients were shown to express higher level of CD46 than CD138^-^ normal counterpart (111).

In terms of safety and efficacy, first phase I study reported for an oncolytic MV (oMV) in 5 patients with cutaneous T-cell lymphomas demonstrated that five of the six injected lesions exhibited tumor regression and partial regression of the distant noninjected lesions in 2 patients with no adverse events higher than grade 1 being observed even with the highest dose of 1,000 TCID_50_ (109). The regression of both injected and noninjected lesions suggest that oncolytic effect by the virus and potential induction of systemic antitumor immunity. Other evidence like elevated serum IL-2, IL-12, and IFN-γ expression level and elevated intratumoral infiltration of CD8^+^ T cells in the injected lesions also suggest induction of pro-inflammatory changes in the patients following oMV administration. Still, these findings should be interpreted with caution as patients were treated with systemic INF-α therapy (113), which could also induce pro-inflammatory changes, to minimize oMV activity in normal tissues of immune-compromised lymphoma patients prior to oMV administration.

More recently published clinical studies utilized oMV expressing either soluble extracellular domain of human carcinoembryonic antigen (CEA; MV-CEA) or human thyroidal sodium-iodide symporter (NIS; MV-NIS) to monitor real-time viral gene expression *in vivo* (110–112). In phase I trial evaluating intraperitoneally administered MV-CEA (10^3^ to 10^9^ TCID_50_) was shown to be well-tolerated in platinum- and paclitaxel-resistant ovarian cancer patients with only one grade 3 arthralgia being observed in one patient (NCT00408590). Viral kinetics could be monitored by increased CEA level in peritoneal fluid in high dose cohort (one patient from 10^8^ and two patients at 10^9^ TCID_50_) and dose-dependent objective response was observed with best objective response of stable disease being observed in 9 out of 9 patients at the dose level of 10^7^ – 10^9^ TCID_50_ while only 5 out of 12 patients achieved stable disease at dose level of 10^3^ – 10^6^ TCID_50_. The median overall survival of the patients receiving MV-CEA was 12.15 months, which is greater than expected median survival of 6 months in similar historical patient cohort. Although immune stimulatory aspect of MV-CEA was not examined in detail in this study, there was no changes in CD4 and CD8 levels following MV-CEA administration and further evaluation of the product in the scope of IO will be needed. Unfortunately, only one other phase I clinical trial utilizing MV-CEA has been completed in patients with recurrent glioblastoma (NCT00390299) and there is no ongoing studies utilizing MV-CEA, thus its immune regulatory properties will likely remain unknown.

Currently, majority of the ongoing clinical trials are utilizing MV-NIS construct rather than MV-CEA, possibly due to NIS having greater clinical applicability as MV-NIS could enhance the accumulation of therapeutic radioisotopes at the tumor lesions and induce additional antitumor effect in preclinical models (114). A phase I/II trial evaluating IV administered MV-NIS either in combination with or without cyclophosphamide (the drug was included to attenuate antiviral immune response) in patients with advanced multiple myeloma (NCT00450814) demonstrated that MV-NIS monotherapy was well-tolerated up to the dose of 10^11^ TCID_50_ with no dose limiting toxicities being observed (111, 112). In terms of efficacy, one patient who received 10^11^ TCID_50_ achieved durable and long-lasting complete response and >25% reduction in serum free light chain levels (a biomarker of plasma cell malignancy like multiple myeloma) being observed in four other patients out of 32 patients. Unfortunately, the uptake of ^123^I was positive in the tumor deposits of only four patients with modest uptake being observed in fraction of the lesions, suggesting virus-induced NIS expression at the current level would not be sufficient to induce radioisotope-mediated antitumor effect. A more in-depth immune profiling of 10 patients who were treated with 10^11^ TCID_50_ revealed 8 out of the 10 patients who did not clinically respond to MV-NIS therapy also exhibited negligible increase in cytotoxic T cell response from the baseline observed prior to virotherapy (112). In general, the patients showed elevated CD8^+^ T cell count in the PBMC and increased proportion of both effector memory and central memory CD8^+^ T cell population following MV-NIS treatment, providing preliminary evidence of pro-inflammatory reaction following systemic virus administration. Increased PD-1 expression level was also observed in CD8^+^ T cell following virus administration, which suggests that MV-NIS in conjunction with ICI may enhance clinical response in patients. Unfortunately, only trial registered to evaluate MV-NIS with ICI has been terminated due to low recruitment (NCT02919449).

Although several phase I or II clinical trials are either active or recruiting for evaluation of MV-NIS in wide-range of cancer types (NCT02364713, NCT01846091, NCT02962167, NCT02700230, and NCT03171493), a clinical trials that focuses on IO property of oMV are needed in the future. Currently, there is only a single ongoing clinical trial that utilizes oMV that expresses pro-inflammatory transgene, Helicobacter pylori Neutrophil-activating Protein (NAP; MV-s-NAP), for the treatment of patients with invasive metastatic breast cancer (NCT04521764). Although preclinical models have demonstrated MV-s-NAP to induce pro-inflammatory response (115), its immune regulatory properties in patients has not been reported to date.

#### 2.4.3 Oncolytic picornaviruses

Currently, two different oncolytic picornaviruses, lerapolturev (previously known as PVSRIPO) and V937 (previously known as CAVATAK and CVA21) are under active clinical development. Another oncolytic picornavirus NTX-010, a Seneca Valley virus, has conducted one phase I trial and II trial reported to date (NCT01048892 & NCT01017601, respectively), but will not be discussed in this section (116). This is due to phase II trial in patients with small cell lung cancer leading to early termination of the trial due to NTX-010 treatment failing to improve overall survival or progression free survival rate compared to the placebo group and early termination of the trial, and no subsequent clinical trial being conducted since the failure (116). Both lerapolturev and V937 natively have a tropism that may be beneficial for cancer therapy application and demonstrated promising therapeutic efficacy in early phases of clinical trials with good safety record, thus these two viruses will be reviewed in greater detail.

Lerapolturev is a genetically modified attenuated version of the poliovirus type 1 Sabin that had its internal ribosome entry site (IRES) replaced with IRES of human rhinovirus type 2 to ablate neurovirulence (117, 118), internalizes into cells *via* CD155, which is upregulated in solid tumors and APCs. Importantly, the infection of APC with lerapolturev has been shown to be nonlethal and reported to induce sustained proinflammatory response and activation of APC (118, 119), which could be beneficial for the instigation of tumor-specific immune response. In support, a phase I clinical trial result evaluating intratumorally administered lerapolturev in patients with unresectable and PD-1 ICI treatment-refractory melanoma revealed that one patient (Patient #11) who was negative for CD155 in pretreatment tumor biopsy (biopsy had small area of viable tumor on the slide, but rather contained abundant CD155^+^ abundant pigment-laden macrophages) showed partial response to treatment per immune-related response criteria (irRC), suggesting that antitumor response may have been achieved *via* infection of immune cells in the tumor microenvironment (120). Overall, the intratumoral administration of lerapolturev led to objective response in 33% of the patients (4 out of 12) who were administered with three doses of lerapolturev in the lesions with tumor regression being observed 10 days after the virus administration. Two patients showed pathological complete responses in both the injected and non-injected lesions with post treatment biopsy samples at the injection site showing abundant macrophage accumulation. Notably, 6 out of 12 patients resuming ICI therapy after lerapolturev treatment had durable disease control and remained progression free at a median follow-up period of 18 months, which suggests potential resensitization of PD-1 ICI refractory tumors to PD-1 blockade. Building on this promising results, multicenter phase II trial evaluating lerapolturev in patients with confirmed PD-1 ICI refractory melanoma with or without pembrolizumab is now ongoing (NCT04577807).

Another phase I study evaluating the convection-enhanced infusion of lerapolturev directly into the tumor tissues in 61 patients with recurrent World Health Organization grade IV glioma also yielded promising results without any sign of neurovirulence symptoms (encephalomyelitis, poliomyelitis, and meningitis) typically associated with wild-type polio infection (118). The overall survival rate was 21% in lerapolturev-treated patients at 24 and 34 months after virus administration and this was higher compared with 14% and 4% survival rate expected in the historical control group at the same timepoint. Eight patients had a durable radiographic response in the lerapolturev-treated tumor with two patients having complete response and surviving for 15.1 and 70.4 months at the time of last follow-up prior to publication of the study and three patients achieving stable to partial radiographic response for 26 to 60 months. A transcriptomic analysis of lerapolturev-treated patient biopsies revealed that very low tumor mutation burden is associated with longer survival after lerapolturev treatment in recurrent glioblastoma patients, likely due to recurrent glioblastoma with lower tumor mutation burden exhibiting enrichment of inflammatory gene signature. Further, anti-PD-1 ICI in recurrent glioblastoma patients also achieved better survival rate in patients with lower tumor mutation burden, suggesting that lerapolturev in combination with PD-1 ICI could be beneficial in similar subset of patients.

Collectively, the results from phase I trials of lerapolturev as monotherapy have provided preliminary clinical evidence that lerapolturev in combination with ICI could be synergistic as lerapolturev may either resensitize the PD-1 ICI refractory tumors or be beneficial in recurrent glioblastoma patients with low tumor burden. Currently, several phase I/II or II trials evaluating lerapolturev in combination with ICIs are ongoing; phase II trials in combination with anti-PD-1 ICI in patients with recurrent glioblastoma (NCT04479241) or PD-1 refractory melanoma (NCT04577807) and phase I/II trial in combination with Anti-PD-1 or PD-L1 ICI in patients with advanced solid tumors (NCT04690699). The interim results from these trials are awaited.

V937, a wild-type coxsackievirus A21, is another oncolytic picornavirus that is under active clinical development and intrinsically possesses tropism to cells expressing intracellular adhesion molecule-(ICAM)-1 and decay-accelerating factor (DAF) (120, 121). This native tropism is beneficial for cancer therapy, as (1) both ICAM-1 and DAF are known to be overexpressed in several cancer types and (2) increased expression of ICAM-1 correlates with metastatic progression of multiple cancers (122–127). Due to this native tropism favoring infection of tumor cells by V937, no additional genetic engineering was performed to attenuate the virulence of the virus or enable cancer-specific replication of the virus.

Lack of additional safety measure other than ICAM-1- and DAF-targeted tropism of the virus could be a safety concern, as ICAM-1 and DAF are both expressed in normal tissues, which could lead to off-target cytolytic effect and adverse events (121, 128, 129). Still, two recently published phase I and phase II study results demonstrated that locoregional (intravesical or intratumoral) administration of the virus in patients with non-muscle-invasive bladder cancer (NMIBC; NCT02316171) or unresectable melanoma (NCT01227551 & NCT01636882), respectively, was well-tolerated with no grade 2 or higher virus-related adverse events observed (120, 127). Further, IV administration of V937 up to 1 × 10^9^ median tissue culture infectious dose (TCID_50_) in patients with advanced cancer was reported to be safe with no grade 3 or 4 product-related adverse events (NCT02043665) (130). These findings suggest that the tropism-mediated cancer specificity of V937 was sufficient to ensure safe locoregional and systemic delivery of the virus to patients, despite normal tissues also expressing its entry molecules ICAM-1 and DAF.

In terms of efficacy, intravesical administration of V937 led to increased surface hemorrhage and inflammation of the tumors and one case of complete tumor regression from 15 NMIBC patients enrolled in the phase I trial (NCT02316171). ICAM-1 expression level in the tumors was shown to correlate with higher virus infectivity and no virus was detectable by IHC in adjacent stromal areas; another entry molecule DAF was also expressed at a high level across all tumor biopsy of patients, showing that V937 infection/replication was dependent on high level of ICAM-1 and DAF expression in NMIBC tumors. V937 treatment led to higher level of high mobility group box 1 (HMGB1) in the urine samples and cytosolic localization of the HMGB1 in the tumor tissues than the paired untreated NMIBC patient samples, suggesting V937-mediated induction of immunogenic cell death. Further, V937-treated tumors exhibited high level of perforin (127, 131), which is indicative of immune cell activation, likely due to elevation of CXCL9 and CXCL10 expression level following virus administration. V937 treatment led to upregulation of immune checkpoint or immunosuppression-related molecules (PD-L1 and LAG3), suggesting that the combination with ICIs targeting these immune checkpoint axes could be beneficial to boost the antitumor immunity of the V937. Phase II clinical trial of V937 in 57 patients with unresectable melanoma also yielded promising therapeutic outcome with 12-month PFS of 32.9% and durable response rate of 21.1%, ultimately resulting in 75.4% of overall survival at 12-month follow-up (NCT01227551 & NCT01636882). Notably, more than 30% reduction in tumor volume at the noninjected lesions at distal metastases sites (lung or liver) were observed in 4 out of the 13 visceral lesions from eight patients, demonstrating that V937 induced systemic antitumor immune response.

In lieu of these immune stimulatory properties of V937 and elevation of immune checkpoint molecules following virus administration, several phase I, I/II, or II trials evaluating the combination of V937 with pembrolizumab or ipilimumab have been either completed (pembrolizumab: NCT02043665, NCT02565992 and ipilimumab: NCT03408587, NCT02307149) or ongoing (pembrolizumab: NCT02824965, NCT04152863, NCT04152863, NCT04303169). Although detailed or final results of the completed or ongoing studies have not yet been published, the interim results reported from some of these trials seem promising: (1) phase 1b trial of V937 in combination with pembrolizumab reported objective response rate of 100% 5 out of 5 evaluable patients with stage IVM1c melanoma and overall objective response rate of 73% out of the 11 patients in 2017 (132) and (2) phase 1b trial in combination with ipilimumab yielding median overall survival of 45.1 months with objective response rate of 30% and median duration of response of 8.8 months in patients with advanced melanoma (133) with manageable serious adverse events in both trials.

In sum, those oncolytic picornaviruses (lerapolturev and V937) have shown promising efficacy in clinical trials both as a monotherapy and as a combination therapy with ICI, showing strong indications of robust antitumor immune response activation by both viruses. Although detailed and finalized study results from the combination therapy trials are not yet available, the interim results are promising and continued clinical development seems warranted.

## 3 OV in combination therapy regimen

Ideally, a new therapeutic modality is expected to improve the therapeutic outcome when used in conjunction with standard care, and at the least, the combination therapy should not be antagonistic. Based on these premises, the next part of this review will explore how OVs can improve the therapeutic potential of standard treatments such as radio-, chemo-, and immunotherapy in both preclinical and clinical studies.

### 3.1 OV in combination with radiotherapy

Radiotherapy, along with surgery, remains the preferred treatment for locoregional tumors, especially in early stages of cancer (134). Radiation regimens have improved and matured over time, leading to improved disease management and patient outcome. Despite these improvements, a locoregional anticancer effect exerted by radiotherapy limits its efficacy in advanced and metastatic stages of the disease (135). Additionally, locoregional tumor recurrence remains a major challenge for efficient disease management by localized cancer therapeutics (136, 137). To this end, OVs that exert the most potent anticancer effect *via* intratumoral administration could be a promising addition to address these limitations of conventional locoregional therapies. In support, several combination strategies of OV with radiotherapy have demonstrated promising therapeutic outcome.

Synergism of the combination of oAd with radiotherapy has been investigated during the last two decades (138, 139), and several oAds in combination with radiotherapy are being evaluated in phase I and II clinical trials (140, 141). One of the main mechanisms of synergism of the combination therapy involves upregulation of transgene expression by radiation through increase in oAd replication (142, 143). Particularly, radiation has been shown to increase cellular internalization of Ad (142), likely due to radiation-induced CAR, integrin, and dynamin 2 expression levels that are integral to endocytosis of Ads (142, 144–148). Alternatively, through preclinical studies, several oAds in combination with radiation have been shown to promote a pro-apoptotic effect in tumor cells over individual therapies (149–153). Additionally, the Ad E1A gene has been shown to sensitize cancer cells to DNA-damaging agents like radiation (149, 154), and deletion of the E1B 19 kDa gene, a homolog of anti-apoptotic Bcl-2-related protein, enhanced the induction of apoptosis in tumor cells in combination with radiation (153).

In support of these preclinical results, a phase I clinical trial examining the combined therapeutic effect of oAd expressing dual suicide genes (Ad5-yCD/mutTKSR39rep-ADP) in combination with intensity-modulated radiotherapy (IMRT) against newly diagnosed intermediate- to high-risk prostate cancer yielded promising outcomes (141). In detail, patients received intraprostatic injections of Ad5-yCD/mutTKSR39rep-ADP (10^11^ VP and 10^12^ VP on Days 1 and 22, respectively), each followed by a 2.6-week cycle of 5-fluorocytosine + ganciclovir prodrug therapy and concomitant 74 Gy IMRT. The combination therapy led to lower tumor positivity in biopsies performed during follow-up (at 6, 12, and 24 months) with respect to historically matched patients who underwent only radiotherapy. Specifically, more than 40% of intermediate- to high-risk patients in the historical cohort tested positive for adenocarcinoma during post-treatment biopsy when treated with radiotherapy alone. On the other hand, only 22% of the evaluable patients receiving combination therapy were positive for adenocarcinoma. More notable therapeutic benefit was achieved by combination therapy in the intermediate-risk group: ≥30% positivity in biopsy was expected in the historical cohort following radiation monotherapy, but none of the 12 intermediate-risk patients were positive for tumor during the last biopsy following combination therapy. None of these 12 intermediate-risk patients (0%) exhibited prostate specific antigen (PSA) relapse during the follow-up period (12 - 48 months). In contrast, frequency of positive biopsy in high-risk patients following combination therapy (45%) did not differ statistically from the expected result (56%) for this prognostic risk group. In terms of safety, the combined treatment did not increase any adverse effects compared with side-effects induced by either monotherapy examined in separate trials or historically. However, no dose-limiting toxicities or treatment-related serious adverse events have been recorded. Overall, this clinical study showed that the combined treatment of oAd and radiation can be beneficial toward improving therapeutic outcomes of prostate cancer patients with no additional safety hazard.

One clinical study examining replication-incompetent Ad in combination with radiation provided some evidence that this combination strategy induces a favorable antitumor immune response. A phase I trial combining replication-incompetent Ad in combination with radiation has been shown to elevate HLA DR^+^ CD8^+^ and CD4^+^ T cell levels in combination therapy compared to radiation monotherapy, suggesting development of a Th1 immune response favorable for IO application (155). Another preclinical study provided further evidence that the combination of oAd and radiation could exert synergistic antitumor effect *via* robust activation of immune cell infiltration (156). Specifically, oAd co-expressing GM-CSF and IL-12 in combination with radiation was shown to inhibit primary tumor growth and its lung metastasis. Importantly, CD4^+^, CD8^+^, and CD11c^+^ immune cell infiltration into tumor tissues was significantly improved in combination therapy with respect to radiotherapy alone. These clinical and preclinical data support the combination of oAd and radiation by exerting a potent antitumor immune response in future clinical trials.

The oHSVs in combination with radiation have been shown to elicit more potent anticancer effect than either treatment administered alone (40, 157, 158). Several mechanisms behind additive or synergistic tumor growth control *via* combination of oHSV and irradiation have been proposed. For example, Mehzir et al. demonstrated that oHSV with γ_1_34.5 gene deletion in combination with ionizing radiation (IR) elicited a more potent anticancer effect than the respective monotherapies due to irradiation-mediated improvement in viral production (159). Their findings demonstrated that γ_1_34.5-deleted oHSV exhibited poorly sustained synthesis of viral DNA at late stages of the infection cycle compared to wild-type HSV. This restricted viral replication could be overcome by combination with IR; the radiation restored late viral gene expression and replication through activation of the p38 pathway, leading to improved viral replication of γ_1_34.5-deleted oHSV (96). Collectively, their findings showed that p38 activation by irradiation enhanced late viral gene expression that subsequently improved viral replication of γ_1_34.5-deleted oHSVs. In another report, G207 (oHSV deficient in viral ribonucleotide reductase (RR) and γ_1_34.5 neurovirulence protein) in combination with IR resulted in better anticancer effects compared with mono therapy *via* upregulation of cellular RR (160). G207 in combination with radiation elicited dose-dependent and synergistic cytotoxic effects against colorectal cancer cells through radiation-mediated enhancement in viral replication of G207. Similar trends were observed *in vivo*, where G207 in combination with IR induced a more potent tumor-growth-inhibiting effect than did the respective monotherapies. Interestingly, the parental strain of G207 named R3616 that only harbors γ_1_34.5 deletion while the RR encoding gene remains intact failed to induce synergistic killing effect in combination with the same irradiation condition as used with G207. These findings are in disagreement with those observed by Mehzir et al. (161), where irradiation improved the viral replication of γ_1_34.5 gene-deleted oHSV. One plausible explanation is that this could be due to (i) different doses (*in vitro* radiation of 250 rad versus 5 Gy (= 500 rad), or (ii) different cancer cell lines used in the two studies. Nonetheless, these discrepancies indicate that more thorough comparative evaluation be explored in the future to better elucidate how irradiation can improve the efficacy of oHSVs.

Although the main mechanism of synergism during combination therapy using oHSVs and radiation remains elusive, this approach has been evaluated in phase I/II clinical trials (162, 163). A phase I trial of G207 in combination with IR against recurrent and progressive glioma showed that the combination was well-tolerated, and no patients developed HSV encephalitis (163). The patients enrolled in the study did not respond to standard therapy, yet six of nine patients achieved stable disease or partial response, at least, at one time point. Importantly, two patients who underwent retreatment under a compassionate use protocol showed significant radiographic response, showing increase in necrotic tumor region and decrease in tumor mass. Notably, the two patients with most significant radiographic response were HSV-1 seronegative at enrollment, suggesting that the pre-existing neutralizing antibody impedes the potency of locally administered oHSVs. In another phase I/II clinical trial, dose-escalating Imlygic (dose range: 10^6^ to 10^8^ PFU) in combination with chemoradiotherapy (70 Gy/35 fractions with concomitant cisplatin 100 mg/m^2^) for treatment of patients with untreated stage III/IV squamous cell cancer of the head and neck (SCCHN) has been evaluated (163). Their findings revealed that Imlygic in combination with chemoradiotherapy was well-tolerated as no dose-limiting toxicity was observed even with multiple administrations (four administrations over 64 day period). HSV was detected in injected and adjacent un-injected tumor lesions at levels higher than the administered dose, showing efficient replication of Imlygic. Importantly, 82.3% of the treated patients showed tumor response by Response Evaluation Criteria in Solid Tumors (RECIST), and 93% of the patients achieved complete remission at the time of neck dissection, performed 6-10 weeks after completion of combination therapy. Further, no patients developed locoregional recurrence, and disease-specific survival was 82.4% at a median follow up of 29 months, a remarkable achievement compared to the 35-55% of SCCHN patients who develop locoregional or metastatic recurrence within two years of conventional therapy. Together, these results clearly illustrate that the combination of oHSV and IR exerts promising therapeutic effects where locoregional tumor control was critical for patient outcome.

Like other OVs, the precise mechanism of synergism between oVV and radiation remains elusive. In one instance, IR has been shown to upregulate viral genes essential for viral replication, improving overall viral production (88). In marked contrast, others have shown that the synergy behind combination therapy of oVV and IR does not rely on increased viral replication, since IR inhibited JNK signaling and subsequently attenuated viral replication (164). In another report, radiotherapy failed to improve oVV replication. External beam radiation therapy (EBRT) used at clinical dose neither affected GL-ONC1 viability nor accelerated the virus replication. Rather, the combination therapy of EBRT and GL-ONC1 showed a synergistic killing effect due to activation of the apoptosis pathway resulting in delayed tumor growth in an orthotopic sarcoma model. While mice showed survival of 16 and 18 days for EBRT and GL-ONC1, respectively, compared to 12 days for the control group, the combination therapy-treated group showed survival up to 27 days with no toxicity (165). Activation of apoptosis in the combination (GL-ONC1 and X-radiation) group was confirmed in a mouse model of head & neck xenograft tumors. The results showed that X-radiation at clinical dose failed to inhibit virus replication, and the combination was most effective to stop tumor growth (166). Similar results with combination of GL-ONC1 and radiation have been obtained in melanoma, glioma, and sarcoma models (164, 165, 167), leading to a phase I trial (NCT01584284) combining IV-administered GL-ONC1 with standard chemoradiotherapy in head & neck carcinoma patients. Results of this trial also showed that the therapy outcome depends on p16 status. Indeed, after 30 months of follow-up in 19 patients, 7 showed treatment failure and 7 deaths were recorded among p16-negative tumors. In contrast, the five patients with p16-positive tumors were alive and disease-free after 36 months (168). Collectively, these reports suggest that further optimization in dosing regimen for combined treatment of OVs and radiation is necessary to translate promising preclinical outcomes into clinical benefits.

### 3.2 OV in combination with chemotherapy

Unlike surgical resection or radiotherapy, chemotherapy exerts its therapeutic effect in a systemic manner and remains integral in treating cancer patients with disseminated disease. The systemic chemotherapy used as an adjuvant therapy for localized surgical resection has been shown to achieve similar therapeutic outcomes to those achieved by radical resection, as early as 1981 (169, 170). Recent studies revealed that chemotherapeutics are also capable of inducing immunogenic cell death (ICD) of cancer cells (171, 172). There are many factors involved in chemotherapeutics-mediated ICD, such as exposure of calreticulin (CRT) (173, 174), adenosine triphosphate (ATP) (175, 176) and release of high mobility group box 1 (HMGB1) (175, 177). Due chemotherapy is used commonly in conjunction with OVs that require localized delivery to induce a notable antitumor effect. In general, systemically administered OVs in several clinical trials (discussed in greater detail in Section 2) induced suboptimal therapeutic benefit, and delivery of OVs to metastatic sites remains a major challenge. For these reasons, chemotherapy as a systemic adjuvant to localized OV therapy is a topic of clinical interest and under active clinical investigation.

oAds in combination with chemotherapy can induce synergistic anticancer effects through several distinct mechanisms, in which both oAd and chemotherapeutics can function as a potent adjuvant to one another. For example, Ad E1A protein can force the cell cycle into S-phase to sensitize cancer cells to DNA-damaging agents (149, 154, 178). On the other hand, several chemotherapeutic drugs have been reported to enhance the cellular internalization of viruses, their replication inside the cells, and expression of transgenes (179–184). Indeed, oAd that contains E1A but has a double deletion of E1B 19- and E1B 55-genes, in combination with cisplatin exerted enhanced cytolytic and apoptotic activities against a wide range of cancer cell types (32). In clinical trial, patients who received intratumoral Oncorine in combination with platinum-based chemotherapy showed 79% response rate compared to 40% observed in the control arm that lacked virus treatment (185). Based on a phase III clinical trial, in 2006, Oncorine was approved by China’s State Food and Drug Administration for treatment of head & neck cancer in combination with chemotherapy. GM-CSF-expressing oAd (ONCOS-102) in combination with pemetrexed, cisplatin, or carboplatin has been shown to induce synergistic antitumor effects in a malignant mesothelioma model (186). Whereas combination chemotherapy (Pemetrexed + Cisplatin or Pemetrexed + Carboplatin) alone or ONCOS-102 monotherapy showed either no or inadequate tumor suppression in an immune-competent mesothelioma model, the combination of these drugs and ONCOS-102 resulted in a synergistic antitumor effect. Based on these preclinical results, a phase I trial (NCT02879669) to examine ONCOS-102 in combination with first-line chemotherapy in patients suffering from malignant mesothelioma has been initiated.

Despite significant improvements in survival of patients with pancreatic cancers by combination of interferon-alpha (IFN) and chemoradiation in clinical trials (16-36% increase in 2-year survival and 35% increase in 5-year survival), it demonstrated limited overall efficacy due to systemic toxicity of IFN and low intratumoral level of the cytokine (187, 188). To overcome these limitations in therapeutic efficacy and safety issues, oAd expressing hamster IFN (OAd-hamIFN) was tested in combination with chemotherapy and/or radiation in regimens mimicking the IFN-based therapies in a preclinical setting (189). oAd-hamIFN potentiated the cytotoxicity of chemotherapeutic drugs (5-FU, gemcitabine, and cisplatin) to yield enhanced pancreatic cancer cell death in both *in vitro* and *in vivo* experimental settings in a hamster model of pancreatic cancer. Particularly, combining OV therapy with 5-FU showed significant tumor growth inhibition in an *in vivo* immunocompetent hamster model.

In line with these preclinical findings, phase I clinical study evaluating intratumorally administered Ad5-yCD/mutTKSR39rep-hIL12 in combination with chemotherapeutics for the treatment of patients with metastatic pancreatic cancer has demonstrated promising results (NCT03281382). For chemotherapy component of the trial, 5-fluorocytosine (5-FC), which is a prodrug that can be converted to 5-FU by yCD/mutTKSR39rep transgene, in combination with one of the standard of care chemotherapy options for the treatment of pancreatic cancer (FOLFIRINOX or gemcitabine plus nab-paclitaxel) were utilized. The treatment was well-tolerated with only one serious adverse event being observed in one patient from the highest dose level (1 x 10^12^ VP). There were strong evidences of immune activation as the combination therapy elevated the serum levels of pro-inflammatory cytokines and chemokines, like IL-12, IFN-γ, and CXCL10, in a virus dose-dependent manner. Further, Ad5-yCD/mutTKSR39rep-hIL12 treatment elevated the number of proliferating NK and T cells (both CD4^+^ and CD8^+^ subsets) in the PBMC of patients. Interestingly, in the low dose cohort (1 x 10^11^ VP) exhibited elevated Tim3, a T cell exhaustion marker, expression level in NK and CD8^+^ T cell population, whereas the expression level was maintained at a similar level to the baseline observed pretreatment in patients receiving higher doses (3 x 10^11^ or 1 x 10^12^ VP). This finding suggests that prevention of T cell exhaustion requires high level of viral doses and pro-inflammatory cytokine expression level. Additionally, 2 out of 6 patients at the highest dose cohort achieved stable disease in both the virus-treated tumors and metastatic lesions, which indicates induction of systemic antitumor immune response. The highest dose cohort also had median progression free survival period of 10.6 months in comparison to 6.4 or 3.3 months expected in patients who receive FOLFIRINOX or gemcitabine alone. Although no conclusive comparison can be made due to small patient number in early phase clinical trial, the preliminary findings seem encouraging.

Several studies in the last two decades have explored the anticancer activity of numerous combinations of oHSVs and chemotherapeutics (190–194), and many potent combination regimens have been identified. One of the strongest merits of oHSV is that its anticancer effect is not inhibited by genotypic alterations commonly observed in tumors (*e.g.*, p53 and Rb pathways), whereas many of the conventional therapies are nullified by these oncogenic mutations (195). Mechanisms underlying synergistic interactions among different oHSV and chemotherapeutics have been described (196). In general, the synergistic outcome of combination therapy requires that chemotherapy not interfere with replication of oHSV in infected tumor cells. Consistently, enhancement of viral replication has been reported as a common mechanism behind the synergistic anticancer effect (197–200).

Based on these strong lines of evidence supporting synergy between a diverse range of chemotherapeutic drugs and different oHSVs, phase I and I/II trials have been conducted to evaluate the safety profile of these combination therapies (201, 202). In a phase I trial, endoscopic ultrasound (EUS)-guided administration of HF10 (a spontaneously mutated oHSV lacking expression of multiple viral genes, γ_1_34.5, U_L_43, U_L_49.5, U_L_55, and U_L_56) into localized and unresectable pancreatic tumors in combination with chemotherapeutics (erlotinib and gemcitabine) was explored (202). In this study, patients underwent one cycle of erlotinib and gemcitabine treatment followed by intratumoral injections (four repeated administrations of 1 × 10^6^ – 1 × 10^7^ PFU of HF10) *via* EUS guidance on the first day of the second chemotherapy cycle. This combination treatment did not yield any grade III or IV adverse effect, suggesting that addition of HF10 to standard chemotherapy was well-tolerated. Of the nine enrolled patients, three showed partial response, four showed stable disease, and two exhibited progressive disease. Notably, tumor shrinkage in two patients was observed, and the tumors were resected. In both cases, surgically resected patients achieved long-term survival, and significant infiltration of CD4^+^ and CD8^+^ T cells was detected either in fibrosis near the residual cancer cells or in the resected tumor specimen. In one case, invasion to the plexus of the superior mesenteric artery decreased following HF10 administration, and the resected specimen showed 90% reduction in cancer cells with fibrosis. Of note, tumors were considered unresectable at the time of enrollment. These results demonstrate that the combination of chemotherapy and oHSV could exert antitumor immune responses critical in IO applications. Although most reports examining the combination therapeutic index of oHSV and chemotherapeutic drug reported additive or synergistic effects, some studies have reported an antagonistic effect on viral replication (203). Thus, the combination of chemotherapy with oHSV warrants careful optimization of parameters such as sequence and timing of administration as well as transgene and drug selection (196).

Several chemotherapies in combination with oVV have been explored. However, in some cases, the combination has been shown to restrict the therapeutic efficacy of oVV and demands careful selection of drug candidates and dosing regimen. For instance, 5-FU and irinotecan (a topoisomerase inhibitor) have been shown to impede viral replication (204). Despite the attenuation in viral replication, the combination therapy with irinotecan resulted in a significant synergy, with a median survival of 87 days in mice with a colorectal tumor treated with combination vvDD and irinotecan compared to 57 and 48 days for the groups receiving vvDD or chemotherapy alone, respectively. Indeed, the authors showed that the virus-infected cells were arrested in S-phase, where they were more sensitive to the irinotecan. The mechanism of synergy was assigned to the direct killing effect of the vaccinia virus and the early recruitment of macrophages (205). A phase I clinical trial (NCT01469611) using Pexa-Vec showed that administration of multiple doses of oVV was safe with no adverse events other than flu-like symptoms in colorectal cancer patients. Furthermore, IV infusion (3.1 × 10^9^ pfu/kg injected biweekly over 8 weeks) resulted in stable disease in 8 of 9 patients (89%) (206). In view of these pre-clinical and clinical data indicating the synergistic effect of oVV and irinotecan combination, a clinical trial on Pexa-Vec and irinotecan combination on refractory colorectal carcinoma patients (NCT01394939) has been initiated. Despite some oVV and chemotherapy combination therapy regimens achieving promising therapeutic outcome, a recent phase III clinical trial evaluating the combined therapeutic effect of Pexa-Vec with the kinase inhibitor sorafenib failed to show any benefits over sorafenib monotherapy in patients with advanced liver cancer (207). Those results illustrate that there are many obstacles to overcome prior to successful clinical adaptation of various OV plus chemotherapeutic combination therapy regimens.

Paclitaxel, targeting tubulin, also has been investigated as a possible combination therapy component. Although paclitaxel caused reduction in viral replication, G2/M phase cell cycle arrest yielding a 2-fold increase in infectivity of the vaccinia virus was reported. The combination of paclitaxel and IV vvDD led to 50% complete and durable response in mice bearing colorectal tumors. The data were in contrast to a 10% response in the vvDD-alone group and 0% in the paclitaxel-only group (208). Compared to paclitaxel, the effects of other drugs in combination therapy are unclear. Gemcitabine, a pro-drug that acts like a nucleoside analog, failed to show any synergistic effect when combined with GL-ONC1 in pancreatic cancer cell lines (209), while its combination with oVV-Smac (an oncolytic vaccinia virus encoding a caspase activator) effectively reduced tumor volume and enhanced the survival rate of pancreatic-cancer bearing mice (210).

Despite synergistic effects observed in the cases described above, some combinations did not show any enhancement in cancer cell killing. A triple therapy with GL-ONC1, nab-paclitaxel, and gemcitabine did not show any significant increase in cytotoxicity in pancreatic cell lines (209). However, this finding was of special interest for clinical application because the combination of nab-paclitaxel and gemcitabine has been approved. GL-ONC1 possessing 3 transgenes (Ruc-GFP, β-glucuronidase and β-galactosidase) provided the potential to non-invasively monitor tumors (89). In this case, despite not bringing a therapeutic benefit to the actual treatment, it could be used safely to monitor tumors.

Taken together, these results suggest that further optimization regarding detailed mechanism of chemotherapeutics how they improve therapeutic outcome of OV is needed to translate promising preclinical results into clinical benefits.

### 3.3 OV in combination with immunotherapy

For a new treatment modality, such as OVs, to enter the clinical landscape, the product should not impede the therapeutic effect of standard care. Ideally, a new therapeutic modality is expected to improve the therapeutic outcome when used in conjunction with standard care, and at the least, the combination therapy should not be antagonistic. Based on these premises, the next part of this review will explore how OVs can improve the therapeutic potential of immunotherapy.

Despite remarkable success in cancer immunotherapeutics in recent times, immunologically ‘cold’ tumors remain a major obstacle for these innovative drugs. Specifically, a wide range of clinically approved immunotherapeutics, including the chimeric antigen receptor (CAR)-T cells and ICIs, are beneficial in only a small subset of cancer patients, as a large subset of patients with immunologically cold tumor respond poorly to the treatment (14, 211–213). In this regard, OVs with their unique inflammatory properties are investigated as promising adjuvants to inflame ‘cold’ tumors and convert them into ‘hot’ tumors that are more responsive to immunotherapy-induced antitumor immune reaction. This OV-induced immunological conversion of the tumor milieu has been shown to be highly favorable toward maximizing the antitumor immune response of several clinically approved cancer immunotherapies. Additionally, cancer vaccines and OVs have been found to exert lower side-effects in cancer patients than do other systemic immunotherapies (214, 215). Since the increased risk of safety hazard in mono- or combination-therapies of clinically approved immunotherapies is a major concern in IO, the low toxicity profile of OV-combined therapies is highly favorable and is being explored actively in preclinical and clinical studies.

Even though immune checkpoint inhibitors (ICI) have changed the treatment paradigm for many cancers, there is medical unmet need in 70-80% of patients who did not response to ICI treatment (216). Certain cancers have a unique tumor microenvironment that has a relative paucity of infiltrating immune effector cells, creating a “cold tumor” (216). Therefore, strategies to utilize OV for warming cold tumor microenvironments are attractive to increase the effectiveness of ICIs, and thus there are multiple ongoing clinical trials evaluating the combination of oAd with ICI. Several preclinical studies involving oAd-ICIs have revealed promising candidates (217). oAd expressing IL-2 and TNF-α has been reported to yield marked increase in intratumoral CD8^+^ T cells when each of these was combined with an anti-PD-1 antibody compared to virus alone. Furthermore, combination therapy with the anti-PD-1 antibody and viral therapy resulted in statistically significant tumor growth suppression and increase in survival compared to virus monotherapy. A clinical trial employing an oAd encoding TNF-α and IL-2 (TILT-123) in combination with an anti-PD-1 antibody is warranted. Recently, two phase I clinical trials evaluating TILT-123 in combination with ICI (pembrolizumab NCT05271318 or avelumab NCT05222932) have been posted (https://clinicaltrials.gov/).

Penetration of ICI into tumor tissues is limited due to abnormal vasculature, tumor interstitial pressure, and excessive extracellular matrix (ECM) accumulation (218). To overcome these challenges, strategies combine the therapeutic efficacy of oAd (oAd/IL12/GM-RLX), which induces antitumor immune response and ECM degradation in tumor microenvironments, and PD-1 immune checkpoint blockade (αPD-1). The combination of oAd/IL12/GM-RLX and αPD-1 induced effective degradation of the tumor ECM. Further, it enhanced intratumoral infiltration of αPD-1 and activated antitumor immune cells. This strategy elicited a potent and durable antitumor immune response against cold tumors. This is the first study showing that expression of four genes (three immune stimulatory genes and another gene specializing in ECM degradation) by a single oAd can overcome the major limitations of ICI therapies, which has emerged in recent clinical trials, by promoting favorable remodeling of both physical and immunological aspects of the tumor.

Combination of oHSV, HSV1716, and PD-1 blockade significantly prolonged survival compared with PD-1 blockade alone or HSV1716 monotherapy in murine rhabdomyosarcoma models (219). This therapeutic outcome was due to increased tumor infiltration of CD4^+^ and CD8^+^ T cells, but not Tregs. Furthermore, this combination strategy was effective to treat glioblastoma (GBM), which is lethal, highly immunosuppressive, and posited to contain GBM stem-like cells (GSCs) (220). Triple combination of oHSV expressing IL-12 with anti-PD-1 and anti-CTLA-4 cure most mice with GSC-derived orthotopic GBM. This curative therapy is associated with large increases in M1-like macrophages and T effector cells (CD4^+^ and CD8^+^ T cells) and decreases in regulatory T cells. These data suggest the combination of ICI and oHSV as an effective treatment strategy for tumor. Similar trends have been observed in early phase clinical trials evaluating oHSV with ICI. For instance, preliminary phase I/II trial results of RP-1 (an oHSV armed with GM-CSF and a truncated highly fusogenic form of the envelope glycoprotein of gibbon ape leukemia virus (GALV-GP-R) to enhance immunogenic cell death) in combination with nivolumab (NCT03767348) have been demonstrating promising therapeutic efficacy in melanoma and non-melanoma skin cancer patients refractory to ICI therapy. In detail, 13 out of 36 melanoma patients showed therapeutic response and 8 out of 13 patients with non-melanoma skin cancer achieved therapeutic response with 5 of these 8 patients achieving complete response (221). Although many of the phase I or II clinical trials evaluating different oHSVs in combination with ICI has seemed to yield promising results (212, 215), these results should be interpreted with caution due to small sample size of patients in early phases of clinical trials. For instance, T-VEC in combination with pembrolizumab, which yielded promising CR rate of 33% in a phase 1b trial (NCT02263508) (222), failed to demonstrate superior PFS or OR over pembrolizumab plus placebo in a phase III trial. Similarly, phase Ib/III trial of same combination therapy regimen in patients with metastatic head and neck squamous cell carcinoma (HNSCC) also failed to demonstrate superior efficacy over historical HNSCC cohort treated with pembrolizumab monotherapy in phase 1b portion of the trial and phase III was not further pursued (NCT02626000) (35).

Combination treatment of anti-CTLA-4 ICI with an oVV was effective in eradicating tumors and provided extended survival compared to monotherapies (223). Building on these preclinical data, clinical studies of Pexa-Vec in combination with ICIs have been initiated, particularly in view of the termination of phase III trial of Pexa-Vec in combination with sorafenib for liver cancer in 2019. Phase I/II clinical trial of Pexa-Vec in combination with anti-CTLA-4 ICI ipilimumab in patients with metastatic or advanced solid tumors is ongoing (NCT02977156). Another phase I/II study of Pexa-Vec in combination with either anti-PD1 ICI durvalumab alone or in triple combination with anti-CTLA-4 ICI tremelimumab has been ongoing for the treatment of patients with advanced colorectal cancer (NCT3206073). Majority of the newly registered and “recruiting” clinical trials listed in http://clinicaltrials.gov as of July of 2022 aim to maximize the immune stimulatory aspects of oVV; 5 out of 7 trials plan to evaluate oVV in combination with ICI (NCT04301011, NCT05061537, NCT04725331, NCT03954067, and NCT03294083) and 5 out of 7 trials utilize oVV harboring either a single or combination of immune stimulatory transgenes (GM-CSF: NCT05376527 & NCT03294083, anti-CTLA4 antibody + GM-CSF: NCT04725331 (224), Fit3 Ligand + anti-CTLA4 antibody+ IL-12: NCT04301011, and IL-7 + IL-12: NCT03954067 (225)).

The potential benefit of combining cytokine-expressing OV with DCs, efficient and specialized APCs that can stimulate naive and memory T cells, was explored for treatment of established tumors (226). Ad-ΔB7/IL-12/4-1BBL, an oAd co-expressing IL-12 and 4-1BBL, exhibited significantly enhanced IFN-γ expression and antitumor efficacy *in vivo*, suggesting that the antitumor type 1 immune response was significantly activated by co-expression of these transgenes. Moreover, Ad-ΔB7/IL-12/4-1BBL in combination with DCs further enhanced antitumor and anti-metastatic effects by enhancing antitumoral type 1 immune response and suppression of type 2 immune response. Furthermore, oAd co-expressing IL-12 and GM-CSF in combination with DC resulted in strong and synergistic antitumor effects compared to the single treatments (227). Of note, combination of this virus with DC caused upregulation of CCL21^+^ lymphatic vessels in tumor tissues and led to considerable increase in endogenous and exogenous DC in DLN, indicating that oAd expressing the proper cytokine could increase the function of DC by stimulating it to differentiate.

Despite several advantages in using oAd and DC in combination therapy, their limited bioavailability and short half-life in solid tumor are critical drawbacks (228). Development of injectable and biodegradable gelatin-based hydrogel as a matrix to protect oAd and DCs in the hostile tumor microenvironment has been attempted. This matrix was shown to protect therapeutic components in the solid tumor and offered sustained release while preserving biological activity (229). oAd- and DC-loaded hydrogel (oAd+DC/gel) showed significantly greater expression of IL-12, GM-CSF, and IFN-γ than either the single treatment (oAd or DC) or oAd in combination with DC (oAd+DC). Furthermore, efficient activation of both endogenous and exogenous DCs, migration of DCs to draining lymph nodes, and tumor infiltration of CD4^+^ and CD8^+^ T cells was observed. oAd+DC/gel significantly increased tumor-specific IFN-γ-secreting immune cells and attenuated tumor-mediated thymic atrophy, associated with immunosuppression in the tumor microenvironment, compared with oAd+DC. These findings suggested that the gel-mediated co-delivery of oAd and DCs offer potent and prolonged antitumor effects and hence warrant future cancer clinical trials.

As innate immune cells, natural killer (NK) cells are unique and play pivotal functions in cancer immune surveillance. These can eliminate a variety of abnormal or stressed cells without prior sensitization and can preferentially kill stem-like cells or cancer stem cells (230). However, the antitumor response of NK cells faces many limitations, including (i) poor ability of NK cells to reach tumor tissues (231), (ii) changes in NK cell-activating receptors and their ligands in tumors (232, 233), and (iii) tumor microenvironment infiltrating suppressive or tolerogenic macrophages and regulatory T (Treg) cells (234) that inhibit their action. A recent study demonstrated that the combination of NK with oAd enhanced its tumor suppression activity (235, 236). The telomerase reverse transcriptase (TERT)-positive tumor treated with CCL20/IL15-armed oAd that replicated under control of the TERT promoter plus NK showed significantly higher antitumor efficacy than either of the treatments alone and induced tumor-specific cytotoxicity of CTLs. These results demonstrated that immunomodulation of the tumor milieu by cytokine-expressing oAd could induce synergistic antitumor immunity in combination with cell-based immunotherapeutics.

Adoptive or CAR-T cell therapy is a promising candidate for cancer immunotherapy. Clinical trials against B cell malignancies with anti-CD19 CAR-T cells demonstrated remarkable therapeutic efficacy, leading to durable complete remission (237–240). However, CAR-T therapy elicits low therapeutic efficacy against solid tumors, possibly due to T cell hypofunction that hinders T cell infiltration and activity (241, 242). Based on these backgrounds, there has been increasing number of reports investigating various OVs to improve the activity of CAR-T cell therapies (243, 244). For example, oAd armed with the chemokine RANTES and IL-15 has been shown to facilitate migration and survival of CAR-T cells and enhance its cytolytic effect in preclinical setting (245). Further, the oAd expressing an EGFR-targeting bispecific T-cell engager (OAd-BiTE) improved the outcome of CAR-T cell therapy in solid tumors (246). CAR-T cells targeting folate receptor alpha (FR-α) successfully infiltrated FR-α-positive and EGFR-positive SK-OV3 xenograft tumors but failed to induce complete responses. However, BiTEs secreted from infected cells redirected CAR-T cells toward EGFR in the absence of FR-α in tumor, indicating increased BiTE-mediated T-cell activation in tumors. As a result, combination of a BiTE-expressing oAd with adoptive CAR-T therapy improved antitumor efficacy and prolonged survival compared with the monotherapies. In lieu of these trends, there is one phase 1 clinical trial evaluating the combination of oAd with HER2-targeted CAR-T cell is ongoing for the treatment of patients with advanced HER2-positive solid tumors (NCT03740256).

Although there are strong scientific rationales and preclinical evidence that support the combination of OV with CAR-T cells to induce synergistic antitumor efficacy, there are growing evidence that contrarily demonstrate that OV-induced type I IFN response can be detrimental to the functionality of T cells, including CAR-T cells, and induce their apoptotic cell death (48, 247, 248). One promising strategy to circumvent this issue is to infect the CAR-T cells directly with OVs which enables systemic delivery of both CAR-T cell and OV to solid tumors (249). In detail, dual-specific CAR-T cells that recognizes viral antigens of the OVs and EGFR of tumors infected with oncolytic reovirus or vesicular stomatitis virus (VSV) exhibited augmented proliferation and biological function due to stimulation of native T cell receptor by viral epitopes. Notably, systemic treatment with OVs-loaded CAR-T cell led to potent antitumor activity, which could be further enhanced by subsequent IV administration of the OV for immune boosting application. This research demonstrated that CAR-T therapy can be combined with OV therapy as a systemic delivery regimen to activate the TCR and bypass the requirement for lymphodepletion.

Collectively, these studies have demonstrated that OV with immune-modulating therapeutic genes and their combination with immunotherapeutics such as DC, NK, CAR-T, and ICI offer a viable strategy to elicit potent induction of antitumor immune response by improving activation, recruitment, and infiltration of immune cells to tumor tissues.

## 4 Future perspective

Both the preclinical and clinical development of OVs are growing at an exponential rate, as these viruses could potentially enhance the poor efficacy of conventional immunotherapy options against immunologically cold tumors in an increasingly immuno-oncology dominant clinical landscape. Further, OVs with more traditional cancer treatment options, like chemo- and radiotherapy, have also yielded promising results, demonstrating the wide applicability of OVs in combination with standard of care. Still, there are many obstacles and unknowns that require further investigation in both preclinical and clinical setting as not all of the clinical trial results have been positive, as evidenced by some landmark phase III trials (Pexa-Vec in combination with sorafenib and T-VEC in combination with pembrolizumab) failing to meet the primary endpoint of trial design despite promising results from early phases of respective clinical trials. In general, there is great variability in patient outcome following OV treatment and insufficient information regarding biological markers that can predict which patient demographic responds to particular OV therapy. More in-depth profiling of responders and nonresponders to OV therapies will be needed to characterize the limitations of OVs more precisely and strategically overcome these issues. Suboptimal systemic administrability of OVs is another hurdle that must be addressed to effectively treat advanced stages of cancer where metastatic or noninjectable lesions are present. To this end, cell-based carriers (CAR-T and mesenchymal stem cells) or synthetic carriers (nanomaterial, polymers, and liposomes) with tumor homing properties have been shown promising preliminary results in preclinical and clinical setting to enhance tumor accumulation of OVs and warrant further investigation.

## 5 Conclusion

This review highlighted various clinical applications of OVs in both monotherapy and combination therapy applications. Since the first clinical trials using OVs in the 1990s, there have been significant advancements in understanding of OV genetics and biology for development of more potent and safer OVs for clinical application. Currently, numerous OVs are under clinical development by multiple pharmaceutical powerhouses as these viruses have shown strong potential to improve the therapeutic outcome of standard cancer treatment options like radiotherapy, chemotherapy, and immunotherapy. Although most clinical data of OV pipelines investigated in this review originated from phase I/II trials and the interpretation of the efficacy results should be taken with caution due to the small sample sizes, the initial reports are highly promising and strongly suggest that OVs could become a common cancer treatment option in the near future.

Many of clinical trials has been developed OVs in conjunction with another therapy and the numbers in combination trials of OVs with ICI have grown over the past 8 years.

## Author contributions

C-OY and A-RY substantially contributed to the conception and design of the article and interpreting the relevant literature. JH revised it critically for important intellectual content and all authors approved the final version of the manuscript.

## Funding

This research was supported by grants from the National Research Foundation of Korea (2016M3A9B5942352 and 2021R1A2C301016611, C-OY; 2021M2E8A1049151 and 2022R1I1A1A01071162, A-RY).

## Conflict of interest

Authors C-OY and JH were employed by company GeneMedicine Co., Ltd.

The remaining author declares that the research was conducted in the absence of any commercial or financial relationships that could be construed as a potential conflict of interest.

## Publisher’s note

All claims expressed in this article are solely those of the authors and do not necessarily represent those of their affiliated organizations, or those of the publisher, the editors and the reviewers. Any product that may be evaluated in this article, or claim that may be made by its manufacturer, is not guaranteed or endorsed by the publisher.
